# DCLK1‐dependent NF‐κB activation mediates p‐STAT3‐induced osteoarthritis progression

**DOI:** 10.1002/ctm2.70712

**Published:** 2026-06-15

**Authors:** Pengfei Li, Chipiu Wong, Yuqiang Wang, Tongzhou Liang, Yichen Que, Wenjie Gao, Yilin Liu, Bo Gao

**Affiliations:** ^1^ Department of Orthopedic Surgery The First Affiliated Hospital of Zhengzhou University Zhengzhou Henan China; ^2^ Department of Orthopedic Surgery Sun Yat‐sen Memorial Hospital of Sun Yat‐sen University Guangzhou Guangdong China; ^3^ Department of Sports Medicine and Rehabilitation Peking University Shenzhen Hospital Shenzhen Guangdong China; ^4^ Department of Orthopedic Surgery, The Affiliated Qingyuan Hospital (Qingyuan People's Hospital) Guangzhou Medical University Qingyuan Guangdong China

**Keywords:** DCLK1, extracellular matrix metabolism, NF‐κB pathway, osteoarthritis, phosphorylated STAT3

## Abstract

**Background:**

Osteoarthritis (OA) is a widespread degenerative joint disorder marked by irreversible cartilage destruction. While signal transducer and activator of transcription 3 (STAT3) is recognised as a pivotal regulator in its pathogenesis, the downstream regulatory cascades of phosphorylated STAT3 (p‐STAT3) in chondrocyte extracellular matrix (ECM) metabolism remain elusive. Our study sought to identify p‐STAT3‐mediated novel pathways driving OA.

**Methods:**

P‐STAT3 expression was detected in human/mouse OA cartilage. Chondrocyte‐specific STAT3 knockout mice (*Stat3* ^fl/fl^; *Col2a1* ^CreERT2^) were first constructed and used to evaluate OA (spontaneous/surgically induced). RNA sequencing combined with cleavage under targets and tagmentation sequencing (CUT&Tag‐seq) identified p‐STAT3 downstream targets, followed by molecular interaction and pathway validation assays.

**Results:**

P‐STAT3 was significantly upregulated in OA cartilage. STAT3 knockout ameliorated OA progression. Mechanistically, p‐STAT3 directly bound the doublecortin‐like kinase 1 (DCLK1) promoter to activate its transcription. DCLK1 interacted with IKKβ, promoting IKKβ phosphorylation and NF‐κB activation, ultimately upregulating MMP13 and downregulating COL2A1.

**Conclusions:**

This study identifies a novel p‐STAT3/DCLK1/IKKβ/NF‐κB axis that regulates cartilage matrix metabolism and OA progression, suggesting new therapeutic targets for OA intervention.

**Key points:**

- Doublecortin‐like kinase 1 (DCLK1) is identified as a novel phosphorylated signal transducer and activator of transcription 3 (p‐STAT3) transcriptional target.
- A novel p‐STAT3/DCLK1/IKKβ/NF‐κB axis regulates chondrocyte extracellular matrix (ECM) metabolism and drives osteoarthritis progression.
- Genetic and pharmacological translational evidence validates the p‐STAT3/DCLK1 axis as a prospective therapeutic target against osteoarthritis.

## Graphical abstract

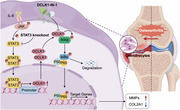

## 1 INTRODUCTION

Osteoarthritis (OA) stands as the primary cause of chronic joint pain and physical disability, affecting more than 300 million individuals globally, particularly the elderly.^1^, ^2^ The pathological hallmarks include gradual degradation of articular cartilage, aberrant subchondral bone turnover and sustained chronic inflammation. Extensive research has focused on the molecular mechanisms driving OA progression.^3^, ^4^, ^5^ Articular cartilage is composed of chondrocytes situated within an extracellular matrix (ECM), and the aberrant expression of crucial ECM metabolic regulators is closely associated with OA onset.^6^, ^7^ Deciphering chondrocyte‐ECM homeostasis networks thus remains a central research priority. A landmark *Nature* study by Choi et al. demonstrated that CH25H and CYP7B1 drive cartilage matrix degradation by upregulating catabolic enzymes, thereby promoting OA pathogenesis.^8^ Serum biomarker studies have also identified MMP13 as correlates of clinical severity.^9^, ^10^, ^11^, ^12^ However, no drugs capable of delaying OA progression have yet been introduced into clinical practice.^13^, ^14^ This gap stems from an incomplete understanding of OA pathogenesis, which has resulted in a lack of predictive biomarkers and therapeutic targets.

Signal transducer and activator of transcription 3 (STAT3), a key effector of the janus kinase (JAK)/STAT pathway, is a critical mediator of inflammation processes and cartilage destruction in OA.^15^, ^16^ Proinflammatory cytokines, including TNF‐α, IL‐1, IL‐6 and IL‐17, induce STAT phosphorylation via JAK activation. Phosphorylated STAT3 (p‐STAT3) binds to specific nuclear DNA response elements to activate target gene transcription,^17^ triggering synovial and cartilage inflammation that drives joint damage.^18^ STAT3 is now recognised to function as a central transcription factor within OA regulatory networks,^19^ underscoring its downstream cascades as critical therapeutic targets.

In OA chondrocytes, p‐STAT3 activates multiple catabolic signalling pathways. The JAK2/STAT3 pathway has been shown to crosstalk with MAPK signalling to amplify chondrocyte inflammation and ECM catabolism.^20^ Additionally, JAK2/STAT3 signalling functionally intersects with the PI3K/Akt pathway, which modulates chondrocyte metabolism and survival.^21^ While reciprocal transcriptional regulation between STAT3 and NF‐κB is established, it remains unknown whether p‐STAT3 directly transcriptionally activates upstream components of the NF‐κB cascade in OA chondrocytes.

Doublecortin‐like kinase 1 (DCLK1), a member of the microtubule‐associated serine/threonine kinase family, was originally identified for its role in mediating microtubule polymerisation and neuronal migration in the nervous system.^22^, ^23^ Outside the nervous system, DCLK1 has been identified as a stem cell marker in the gastrointestinal tract and pancreas.^24^, ^25^ Emerging evidence indicates a significant role for DCLK1 in inflammation‐related diseases. For instance, Kim et al. found that DCLK1 fosters colorectal cancer progression by shaping the inflammatory tumour microenvironment.^26^ Roy et al. identified the Notch‐DCLK1 pathway driving colitis development in mouse models.^27^ Huang et al. revealed that macrophage‐derived DCLK1 accelerates atherosclerosis via IKKβ binding and subsequent induction of inflammatory responses.^28^ Furthermore, Luo et al. described a novel mechanism wherein DCLK1 directly binds IKKβ to activate NF‐κB pathway, highlighting its therapeutic potential for inflammatory diseases.^29^ Given that aberrant NF‐κB activation drives OA cartilage degeneration via oxidative stress and inflammation, the function of DCLK1 in OA pathogenesis remains to be elucidated.

Here, we demonstrate that p‐STAT3 is upregulated in OA cartilage from both humans and mice, and chondrocyte‐specific STAT3 knockout attenuates both spontaneous and destabilisation of the medial meniscus (DMM)‐induced OA. Using integrated RNA sequencing (RNA‐seq) and cleavage under targets and tagmentation sequencing (CUT&Tag‐seq), we identify DCLK1 as a direct transcriptional target of p‐STAT3. Mechanistically, DCLK1 binds IKKβ to enhance its phosphorylation and subsequent NF‐κB activation, leading to ECM dysregulation. Finally, we show that pharmacological blockade of DCLK1 with DCLK1‐IN‐1 alleviates OA progression in mice. Our findings define a novel p‐STAT3/DCLK1/IKKβ/NF‐κB axis and establish DCLK1 as a promising target for OA intervention.

## 2 MATERIALS AND METHODS

### 2.1 Human specimens collection

Human articular cartilage specimens were sourced from OA patients undergoing total knee arthroplasty and from patients with joint trauma but no history of joint disease. The research was approved by the Clinical Trials Ethics Committee of the First Affiliated Hospital of Zhengzhou University. Detailed patient data are listed in Table S1.

### 2.2 Experimental animals and animal models

#### 2.2.1 Mouse strains

The *floxed*‐Stat*3* (*Stat3* ^fl/fl^) mice were obtained from The Jackson Laboratory (Stock No.: 016923). The *Col2a1* ^CreERT2^ mice were kindly provided by Professor Peiqiang Su of Sun Yat‐sen University. *Stat3* ^fl/fl^; *Col2a1* ^CreERT2^ mice were produced by crossbreeding *Stat3* ^fl/fl^ with *Col2a1* ^CreERT2^ mice. Genotypes were confirmed by PCR using tail genomic DNA and the primers shown in Table S2. All mice were housed in a specific pathogen‐free animal facility. All experiments were restricted to male mice in order to reduce animal usage. Approval for the animal procedures was obtained from the Institutional Animal Care and Use Committee of Sun Yat‐sen University.

#### 2.2.2 Tamoxifen induction

*Stat3* ^fl/fl^; *Col2a1* ^CreERT2^ mice were administered 100 mg/kg of tamoxifen (TAM; Sigma) intraperitoneally (i.p.) for 5 consecutive days to induce Cre‐mediated *Stat3* knockout (subsequently designated as cKO). Littermates of the same genotype injected with corn oil alone served as wild‐type (WT) controls.

#### 2.2.3 DMM‐induced OA model

To induce OA, 8‐week‐old cKO and WT littermates underwent DMM operations. In brief, following anaesthesia, the right knee was disinfected for aseptic operation. The medial meniscus was rendered unstable by transecting the medial menisco‐tibial ligament. Sham‐operated mice underwent skin and muscle incision without ligament transection and served as controls. Mice were euthanised 8 weeks post‐surgery.

#### 2.2.4 AAV‐mediated DCLK1 overexpression

AAV9‐NC (negative control) and AAV9‐Dclk1 vectors were purchased from Tsingke. For in vivo overexpression, anaesthetised mice were administered a single intra‐articular dose of AAV (5 × 10^9^ vg [viral genomes] in 10 µL) once using 34 gauge (G) needles (Hamilton) 1 week after surgery.

#### 2.2.5 DCLK1 inhibition experiment

DMM procedures were conducted on 10‐week‐old WT mice. Four weeks post‐surgery, the mice received 10 mg/kg of DCLK1‐IN‐1 (MedChemExpress) daily for 4 consecutive weeks. The control group was administered vehicle (.5% DMSO in saline). Mice were euthanised 8 weeks post‐surgery.

### 2.3 Isolation and culture of primary chondrocytes

Primary chondrocytes were extracted from the articular cartilage of postnatal day 5 (P5) mice. Under a stereomicroscope, cartilage tissue was minced into approximately 1 mm^3^ fragments and then digested with type II collagenase (Invitrogen) at 37°C for 4 h. Filter the cell suspension using the cell strainer. Chondrocytes were maintained in dulbecco’s modified eagle’s medium (DMEM) complete medium (Gibco) and maintained in a 5% CO_2_ humidified incubator at 37°C. All cellular experiments were conducted using chondrocytes at passages 1–2.

### 2.4 Quantitative real‐time PCR

Whole cellular RNA was isolated from chondrocytes with TRIzol reagent and reverse‐transcribed into cDNA with the PrimeScript RT Reagent Kit (Servicebio). Quantitative real‐time PCR (RT‐qPCR) was then executed on the Roche Light Cycler 480 system with the SYBR Premix Ex Taq Kit (Novoprotein). GAPDH was used for normalisation, and the 2^−ΔΔCt^ method was applied to calculate relative mRNA levels. The primer sequences are provided in Table S3.

### 2.5 Immunoblotting

Cell and tissue lysates were prepared using RIPA buffer (Beyotime) containing a cocktail of protease and phosphatase inhibitors (Servicebio). Immunoblotting was carried out as previously described.^1^ Comprehensive details regarding the antibodies, including suppliers and catalogue numbers, are provided in the Supporting Information.

### 2.6 Immunofluorescence

Chondrocytes were seeded on coverslips. After the indicated treatments, cells were fixed in 4% paraformaldehyde for 20 min. Cells were then permeabilised using .1% Triton X‐100 for 15 min, and blocked with 5% normal goat serum for 1 h to minimise nonspecific background. After blocking was completed, cells were probed with the corresponding primary antibodies and incubated at 4°C for 24 h. This was followed by incubation with fluorophore‐conjugated secondary antibodies (Alexa Fluor 488/594, Servicebio, 1:200) for 1 h in the dark. Mounting was performed using a DAPI‐containing mounting medium (Servicebio), and fluorescence signals were visualised under a fluorescence microscope (Leica).

### 2.7 Histology and immunohistochemistry

Cartilage specimens were fixed with 4% paraformaldehyde for 24 h, followed by gradient dehydration, paraffin embedding and cutting into 5‐µm‐thick sections. Deparaffinised sections were used for both histological assessment (Safranin O & Fast Green, SO&FG) and immunohistochemistry (IHC) detection. For SO&FG (Servicebio), slices underwent Safranin O for 10 min, followed by counterstaining with Fast Green for 5 min. For IHC, sections after antigen retrieval were blocked in goat serum and allowed to react with primary antibodies recognising STAT3 (1: 200), p‐STAT3 (1: 200), COL2A1 (1: 100) or MMP13 (1: 200) at 4°C for 24 h. Staining was performed with a 3,3′‐diaminobenzidine tetrahydrochloride horseradish peroxidase chromogenic kit (Servicebio), and images were acquired under a light microscope (Olympus).

### 2.8 Cell transfection and drug treatment

#### 2.8.1 Transfection

Small interfering RNAs (siRNAs) against STAT3 (si‐STAT3) and DCLK1 (si‐DCLK1) were constructed by Tsingke. Briefly, chondrocytes were plated in six‐well plates at 1 × 10^5^ cells per well. When cells reached 70%–80% confluence, transfection was performed using Lipofectamine 3000 (Invitrogen). Culture medium was changed to complete medium at 6–8 h post‐transfection, and cells were maintained for downstream experiments. The siRNA sequences are provided in Table S4.

For adenovirus transduction, replace the culture medium with serum‐free DMEM when the confluence of chondrocytes reaches approximately 80%. Cells were transduced with adenoviruses (Ad‐GFP, Ad‐Cre, Tsingke) at 1 × 10^8^ PFU/mL. After 2 h incubation, we replaced the culture medium with complete medium. The cells were harvested after 48 h for subsequent experiments.

#### 2.8.2 Cell viability assay

Cell viability was analysed with the Cell Counting Kit‐8 (CCK‐8, Beyotime). Chondrocytes were cultured in 96‐well plates at 5 × 10^3^ cells/well and exposed to various doses of IL‐1β (5, 10, 20 ng/mL) or Colivelin (.1, .5, 1, 2 µM) for 24, 48 and 72 h. For each time point, the wells were supplemented with 10 µL of CCK‐8 solution and incubated at 37°C for 2 h. The absorbance was subsequently recorded at 450 nm with a microplate reader, with the DMSO‐treated control group serving as the reference.

#### 2.8.3 Drug treatment

Chondrocytes were treated with IL‐1β (10 ng/mL, Novoprotein) to mimic OA inflammatory conditions, Stattic (1 µM, MedChemExpress) to inhibit STAT3 phosphorylation, Colivelin (.5 µM, MedChemExpress) to activate STAT3 phosphorylation or DCLK1‐IN‐1 (1 µM, MedChemExpress) to inhibit DCLK1 kinase activity. Cells were harvested 72 h after treatment for subsequent experiments.

#### 2.8.4 CRISPR/Cas9‐mediated knockout of DCLK1

To generate DCLK1 knockout chondrocytes, a lentiviral CRISPR/Cas9 vector targeting mouse *Dclk1* (target sequence: 5′‐CTTGGTGACTTGCCCGAGCG‐3′) was constructed using the lentiCRISPR v2‐cmv‐ZsGreen‐GSGP2A‐puro backbone and purchased from Tsingke. Primary mouse chondrocytes were transduced with the lentivirus, followed by puromycin selection (2 µg/mL) for 72 h. Living cells were amplified in culture, and DCLK1 protein ablation was confirmed by immunoblotting before subsequent experiments.

### 2.9 Micro‐CT analysis

After fixation in 4% paraformaldehyde for 24 h, mouse knee joints were conducted to micro‐CT scanning (SkyScan, Bruker). Three‐dimensional reconstructions were generated with NRecon software (Bruker). The following parameters were analysed using CTAn software (Bruker): calcified meniscus volume, synovial tissue volume and bone volume/tissue volume (BV/TV) of the medial tibial subchondral bone.

### 2.10 RNA‐seq

Whole RNA was isolated from chondrocytes (WT vs. STAT3 knockout, treated with IL‐1β) using TRIzol reagent. Post‐quality control, sequencing was undertaken by Seqhealth Technology Co., Ltd. Differentially expressed genes (DEGs) were defined as those with a fold change >2 and *p* < .05. Gene ontology (GO) and Kyoto encyclopedia of genes and genomes (KEGG) pathway enrichment analysis were performed on the DEGs utilising DAVID Bioinformatics Resources 6.8.

### 2.11 CUT&Tag‐seq

Identify DNA fragments bound to p‐STAT3 in chondrocytes using CUT&Tag technology. Sequencing libraries were then constructed using the Hyperactive Universal CUT&Tag Assay Kit for Illumina (Vazyme, TD903). Amplified libraries were quantified and subjected to sequencing by Seqhealth Technology Co., Ltd. Sequence quality was assessed with FASTQC, and the cleaned‐up reads were mapped to the reference genome using BWA. Genome‐wide distribution of signals was visualised with the integrative genomics viewer (IGV). Peaks exhibiting a fold change >2 with a false discovery rate <.1 were classified as differentially enriched, while those without a significant log_2_ fold change were defined as unchanged.

#### 2.11.1 Public ChIP‐seq data analysis

Publicly available STAT3 ChIP‐seq datasets (GEO accession: GSM5188998 and GSM2324099) and H3K27ac ChIP‐seq dataset (GEO accession: GSM7655998) were sourced from the GEO database (https://www.ncbi.nlm.nih.gov/geo/). Quality control metrics and aligned read counts were verified against the original publication for each dataset. The IGV was used to visualise the ChIP‐seq signals at the *Dclk1* locus and identify putative p‐STAT3‐binding sites.

### 2.12 Chromatin immunoprecipitation‐qPCR

Chromatin immunoprecipitation‐qPCR (ChIP‐qPCR) experiment utilised the ChIP Detection Kit (Beyotime). In brief, chondrocytes were crosslinked with 1% paraformaldehyde, followed by lysis and sonication to obtain 200–500 bp fragmented DNA. Protein–DNA complexes were subsequently IP via incubation at 4°C with anti‐p‐STAT3 antibody or IgG as a negative control. Following washing and elution, the precipitated DNA was purified and quantified by qPCR using primers targeting the DCLK1 promoter (Site 1). Primer sequences are provided in Figure 6J.

### 2.13 Dual‐luciferase reporter assay

Clone the DCLK1 promoter region containing site 1 (WT or mutant) into the pGL3‐Basic vector (Tsingke). Chondrocytes were then co‐transfected with the constructed reporter plasmid either a STAT3‐overexpression (OE‐STAT3) or NC plasmid each at 2 µg/mL, with Lipofectamine 3000 reagent. Forty‐eight hours after transfection, luciferase activities were measured using the Dual‐Luciferase Reporter Assay Kit (Beyotime), with Renilla luciferase activity serving as an inner control.

### 2.14 Co‐immunoprecipitation

Cells were lysed in IP buffer (Beyotime) containing protease inhibitors. Equivalent amounts of protein (500 µg) were incubated overnight at 4°C with an anti‐DCLK1 antibody or normal IgG, after which Protein A/G agarose beads (Beyotime) were introduced and further incubation at 4°C for 4 h. After five washes with IP buffer, the captured immune complexes were eluted and detected by immunoblot analysis with anti‐IKKβ or anti‐DCLK1 antibody.

### 2.15 Statistical analysis

All quantitative data are presented as the mean ± standard deviation (SD). Pairwise differences between two independent groups were performed with the unpaired two‐tailed Student's *t*‐tests. When comparing more than two groups under a single independent factor, one‐way ANOVA was conducted, and Tukey's post hoc procedure was applied. Experimental designs incorporating two independent factors were analysed by two‐way ANOVA with Tukey's post hoc correction. All statistical analyses were carried out in GraphPad Prism version 7.0. Differences were considered statistically significant at *p* < .05.

## 3 RESULTS

### 3.1 Phosphorylated STAT3 is significantly upregulated in OA cartilage of humans and mice

**FIGURE 1:**
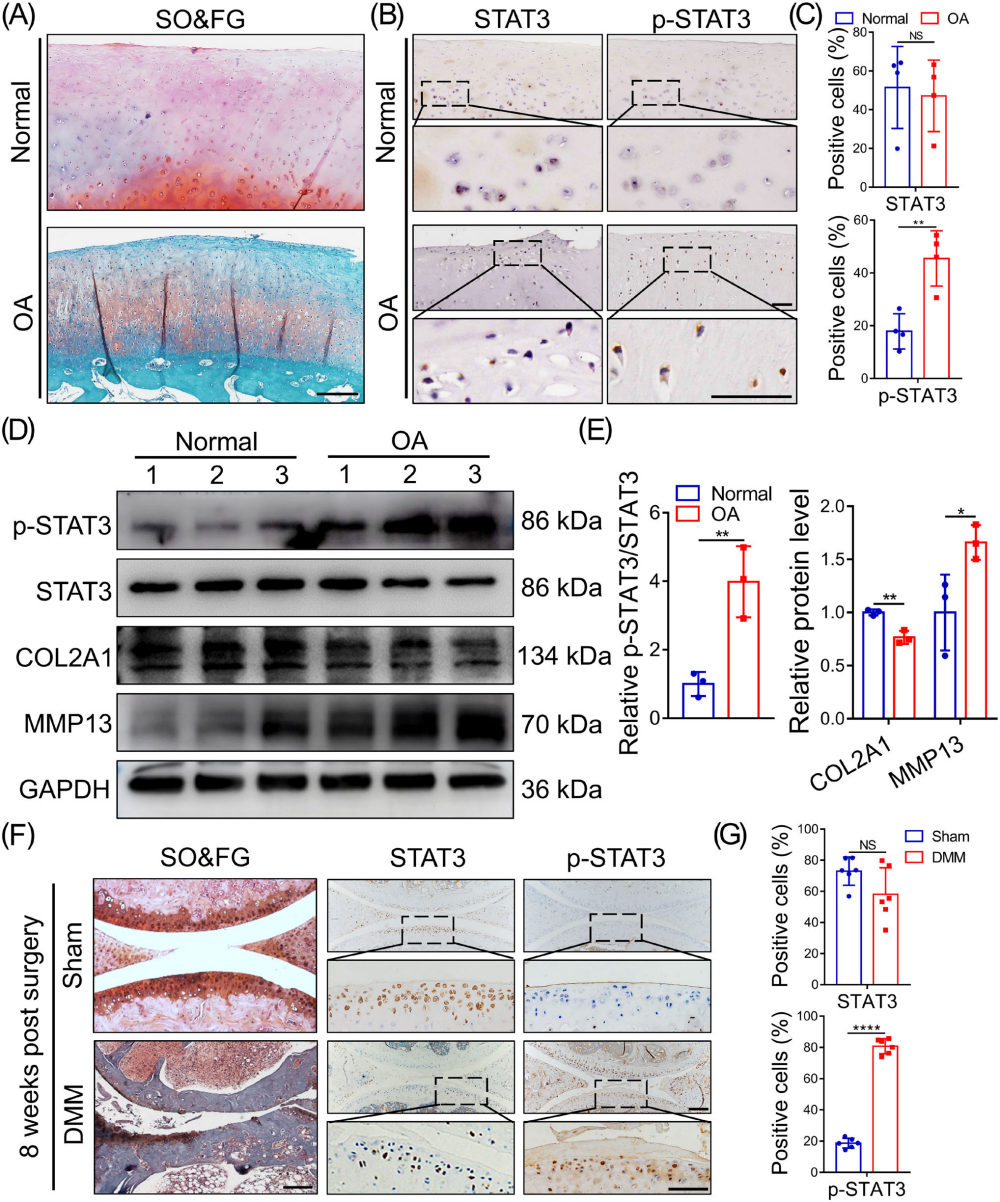
Phosphorylated signal transducer and activator of transcription 3 (STAT3) is significantly upregulated in osteoarthritis (OA) cartilage from both humans and mice. (A) Representative Safranin O & Fast Green (SO&FG) staining images of cartilage from different groups. Scale bars: 200 µm. (B) Representative immunohistochemistry (IHC) staining images of STAT3 and phosphorylated STAT3 (p‐STAT3) in different groups. Scale bars: 50 µm. (C) Quantification of STAT3‐ and p‐STAT3‐positive chondrocytes (*n* = 4 biological replicates). (D, E) Immunoblotting (D) of p‐STAT3, STAT3, COL2A1 and MMP13 in cartilage tissues from normal and OA human knees, with quantitative analysis (E). (F) Representative SO&FG and IHC staining of STAT3 and p‐STAT3 in different groups. Scale bars: 100 µm. (G) Quantitation of STAT3‐ and p‐STAT3‐positive chondrocytes (*n* = 6 biological replicates). NS, not significant. ^*^ *p* < .05. ^**^ *p* < .01. ^****^ *p* < .0001.

As a core transcription factor of the JAK/STAT pathway,^30^ STAT3 expression and activation status in OA pathogenesis require systematic validation. We first quantified p‐STAT3 and total STAT3 levels in human articular cartilage from OA patients and trauma controls. SO&FG staining confirmed severe matrix loss in OA cartilage via reduced safranin O reactivity (Figure 1A). IHC revealed a dramatic increase in p‐STAT3‐positive chondrocytes in OA cartilage, while total STAT3‐positive cell percentages remained comparable between groups (Figure 1B,C), indicating that STAT3 phosphorylation (rather than total STAT3 expression) is specifically induced in OA. This finding was confirmed by immunoblotting, which revealed significantly elevated p‐STAT3 levels in OA cartilage, accompanied by decreased COL2A1 (a major ECM component) and increased MMP13 (a key matrix‐degrading enzyme), with total STAT3 unchanged (Figure 1D,E).

We further examined p‐STAT3 expression in DMM‐induced OA mice. Consistent with human data, SO&FG staining showed marked cartilage damage 8 weeks post‐surgery. DMM mice had significantly more p‐STAT3‐positive chondrocytes in knee joints than sham controls, while total STAT3‐positive cell numbers did not differ (Figure 1F,G). Overall, these data establish that p‐STAT3 is significantly upregulated in both human and mouse OA cartilage and correlates with disease pathology.

### 3.2 Chondrocyte‐specific STAT3 deletion alleviates spontaneous and instability‐induced OA in mice

**FIGURE 2:**
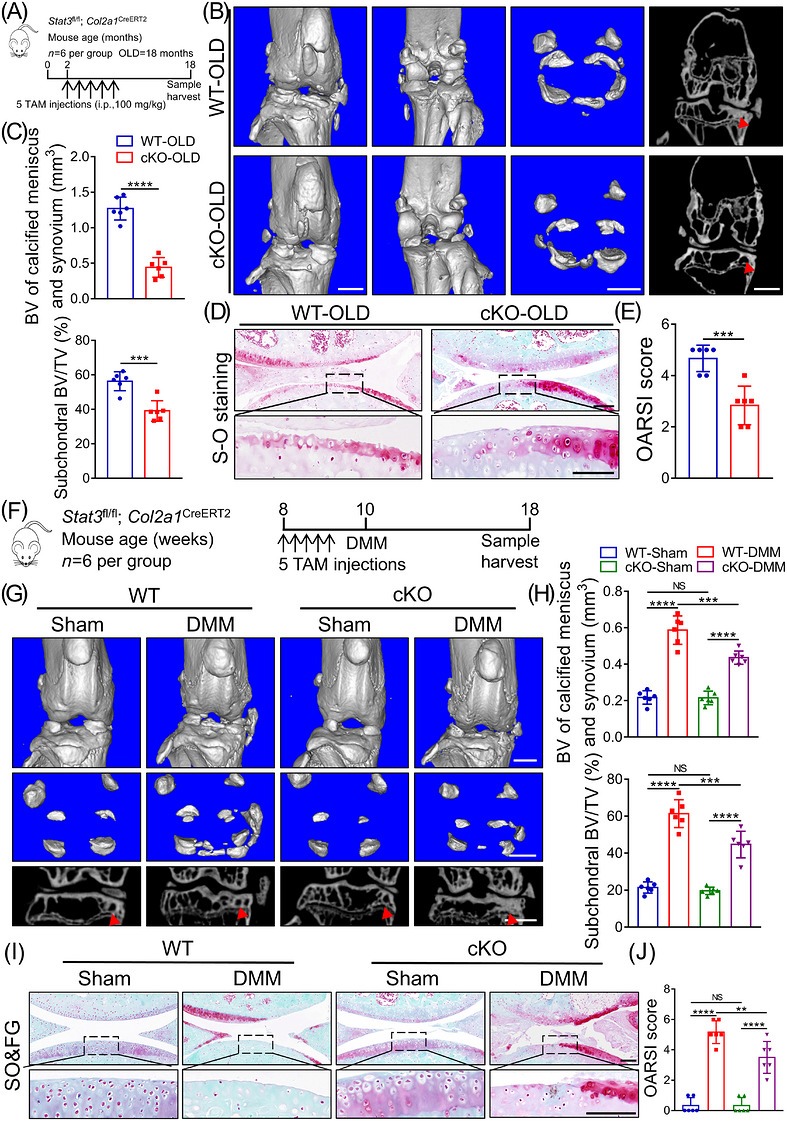
Chondrocyte‐specific signal transducer and activator of transcription 3 (STAT3) deletion alleviates spontaneous and instability‐induced OA in mice. (A) Schematic overview of the experimental protocol. *Stat3* ^fl/fl^; *Col2a1* ^CreERT2^ mice aged 2 months were administered intraperitoneal TAM (cKO, *n* = 6) or corn oil (WT, *n* = 6) for 5 consecutive days. Sixteen months post‐TAM administration, mice were euthanised and knee joint were harvested. i.p., intraperitoneal. TAM, tamoxifen. (B) Three‐dimensional reconstruction from micro‐CT scans of WT‐OLD (18 months) and cKO‐OLD (18 months) knee joints. Red arrows, medial tibial subchondral bone. Scale bar: 1.0 mm. (C) The calcified meniscus and synovial tissue volume, and BV/TV of the medial tibial subchondral bone, were analysed by micro‐CT. BV, bone volume. TV, tissue volume. (D, E) Safranin O & Fast Green (SO&FG) staining of knee joint sections and quantification of Osteoarthritis Research Society International (OARSI) score. Scale bar: 100 µm. (F) Schematic overview of the experimental design. At 8 weeks old, *Stat3* ^fl/fl^; *Col2a1* ^CreERT2^ male mice aged 2 months were administered intraperitoneal TAM (cKO, *n* = 6) or corn oil (WT, *n* = 6) for 5 consecutive days. Two weeks later, mice in the cKO and WT groups underwent DMM or sham surgery, respectively. Knee joints were obtained 8 weeks post‐operation. TAM, tamoxifen. DMM, destabilisation of the medial meniscus. (G, H) Three‐dimensional reconstruction and quantitative analysis from micro‐CT scans. (I, J) SO&FG staining of knee joint sections and quantification of OARSI score. Scale bar: 100 µm. NS, not significant. ^*^ *p* < .05. ^**^ *p* < .01. ^***^ *p* < .001. ^****^ *p* < .0001.

To elucidate the functional role of p‐STAT3 to OA, we generated chondrocyte‐specific STAT3 knockout mice (*Stat3* ^fl/fl^; *Col2a1* ^CreERT2^), with knockout efficiency confirmed by immunoblotting (Figure S1). We first assessed the effect of STAT3 deficiency on age‐related spontaneous OA. TAM was administered i.p. to 8‐week‐old *Stat3* ^fl/fl^; *Col2a1* ^CreERT2^ mice for 5 consecutive days to induce Cre‐mediated *Stat3* deletion, with corn oil‐treated littermates serving as WT controls (Figure 2A).

Micro‐CT analysis revealed severe joint pathology in WT‐OLD mice, including increased calcified meniscus and synovial volume, and elevated BV/TV in the medial tibial subchondral bone. All these abnormalities were significantly ameliorated in cKO mice (Figure 2B,C). Histological evaluation further confirmed improved cartilage integrity in cKO mice, as evidenced by reduced Osteoarthritis Research Society International (OARSI) score and increased cartilage area (Figure 2D,E). Molecularly, cKO cartilage showed increased COL2A1‐positive cells and decreased MMP13‐positive cells (Figure S2A,B), along with reduced p‐STAT3 expression (Figure S3A,B), consistent with the protective effect of STAT3 knockout against age‐related OA.

We next evaluated the impact of STAT3 deletion in a DMM‐induced model. Eight‐week‐old WT and cKO mice underwent sham or DMM surgery, and knee joints were analysed by micro‐CT 8 weeks post‐surgery (Figure 2F). STAT3 deficiency significantly attenuated DMM‐induced OA progression: cKO mice exhibited reduced soft tissue calcification and lower BV/TV (Figure 2G,H), better preserved cartilage matrix, and lower OARSI scores (Figure 2I,J). ECM homeostasis was also restored in cKO mice, with increased COL2A1 and decreased MMP13 expression (Figure S2C,D). Correspondingly, p‐STAT3‐positive chondrocytes were markedly decreased in cKO‐DMM joints (Figure S3C,D), further confirming that STAT3 deletion in chondrocytes retards OA progression.

### 3.3 Phosphorylated STAT3 promotes ECM metabolic dysregulation in chondrocytes

**FIGURE 3:**
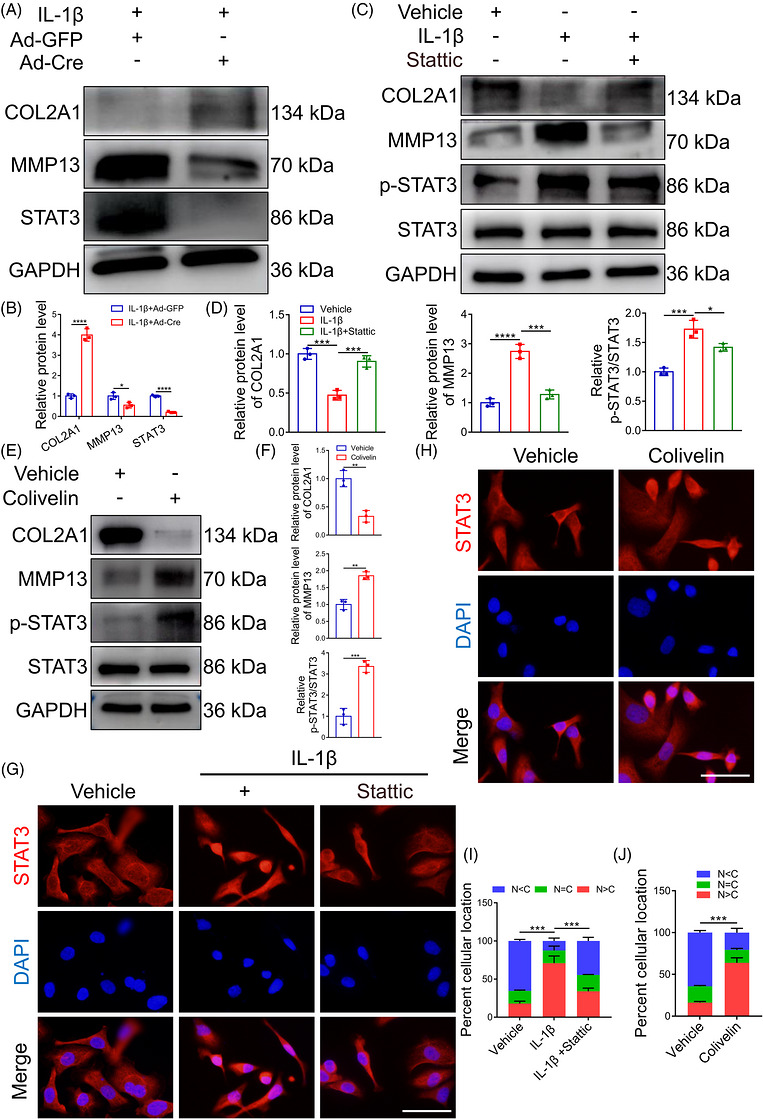
Phosphorylated signal transducer and activator of transcription 3 (STAT3) promotes extracellular matrix (ECM) metabolism disorder in chondrocytes. (A, B) Immunoblotting analysis of COL2A1, MMP13 and STAT3 in primary articular chondrocytes from wild‐type (WT) mice, following transfection with Ad‐GFP or Ad‐Cre and 72 h treatment with vehicle or IL‐1β (10 ng/mL). The band intensities were quantified and normalised against GAPDH. Ad, adenovirus. (C, D) Immunoblotting analysis of COL2A1, MMP13, p‐STAT3 and STAT3 in chondrocytes treated with IL‐1β and/or Stattic (1 µM). The band intensities were quantified and normalised against GAPDH. (E, F) Immunoblotting analysis of COL2A1, MMP13, p‐STAT3 and STAT3 in chondrocytes treated with vehicle or Colivelin (.5 µM). The band intensities were quantified and normalised against GAPDH. (G, H) Representative immunofluorescence (IF) images of the nuclear translocation of STAT3 (red) in chondrocytes treated as indicated. DAPI (blue) stains nuclei. Scale bars: 25 µm. (I, J) Assessment of STAT3 nuclear translocation in chondrocytes treated with vehicle or Colivelin. N < C, cytoplasmic STAT3 exceeds nuclear STAT3. N = C, comparable nuclear and cytoplasmic STAT3. N > C, nuclear STAT3 predominates over cytoplasmic STAT3. ^*^Statistical evaluation focused on comparing the proportion of N > C cells among the groups. Quantitative analyses were based on three biological replicates. ^*^ *p* < .05. ^***^ *p* < .001. ^****^ *p* < .0001.

To directly investigate p‐STAT3 function in chondrocyte ECM metabolism, we performed in vitro studies using primary articular chondrocytes from P5 WT mice, targeting STAT3 expression/activation and detecting key ECM markers via immunoblotting and immunofluorescence (IF). Chondrocytes were transfected with adenoviruses expressing GFP or Cre recombinase, followed by treatment with vehicle or IL‐1β to mimic OA inflammatory conditions. Immunoblotting showed that Ad‐Cre‐mediated *Stat3* ablation reversed IL‐1β‐induced COL2A1 downregulation and MMP13 upregulation (Figure 3A,B). Next, pharmacological inhibition with Stattic (a selective STAT3 phosphorylation inhibitor) similarly reduced p‐STAT3 levels (without altering total STAT3) and normalised IL‐1β‐induced ECM marker dysregulation (Figure 3C,D). Conversely, STAT3 activation with Colivelin increased p‐STAT3 levels (total STAT3 unchanged) and induced ECM catabolism, reducing COL2A1 and elevating MMP13 (Figure 3E,F). IF staining results were consistent with immunoblotting findings. IL‐1β treatment promoted STAT3 nuclear translocation (a hallmark of STAT3 activation), which was blocked by Stattic (Figure 3G,I). Colivelin treatment, by contrast, robustly induced STAT3 nuclear translocation (Figure 3H,J). Importantly, CCK‐8 assays verified that neither IL‐1β (10 ng/mL) nor Colivelin (.5 µM) affected chondrocyte viability under these experimental conditions (Figure S4). Collectively, these data demonstrate that p‐STAT3 directly drives chondrocyte ECM dysregulation in a phosphorylation‐dependent manner.

### 3.4 Phosphorylated STAT3 regulates chondrocyte matrix metabolism by activating the NF‐κB pathway

**FIGURE 4:**
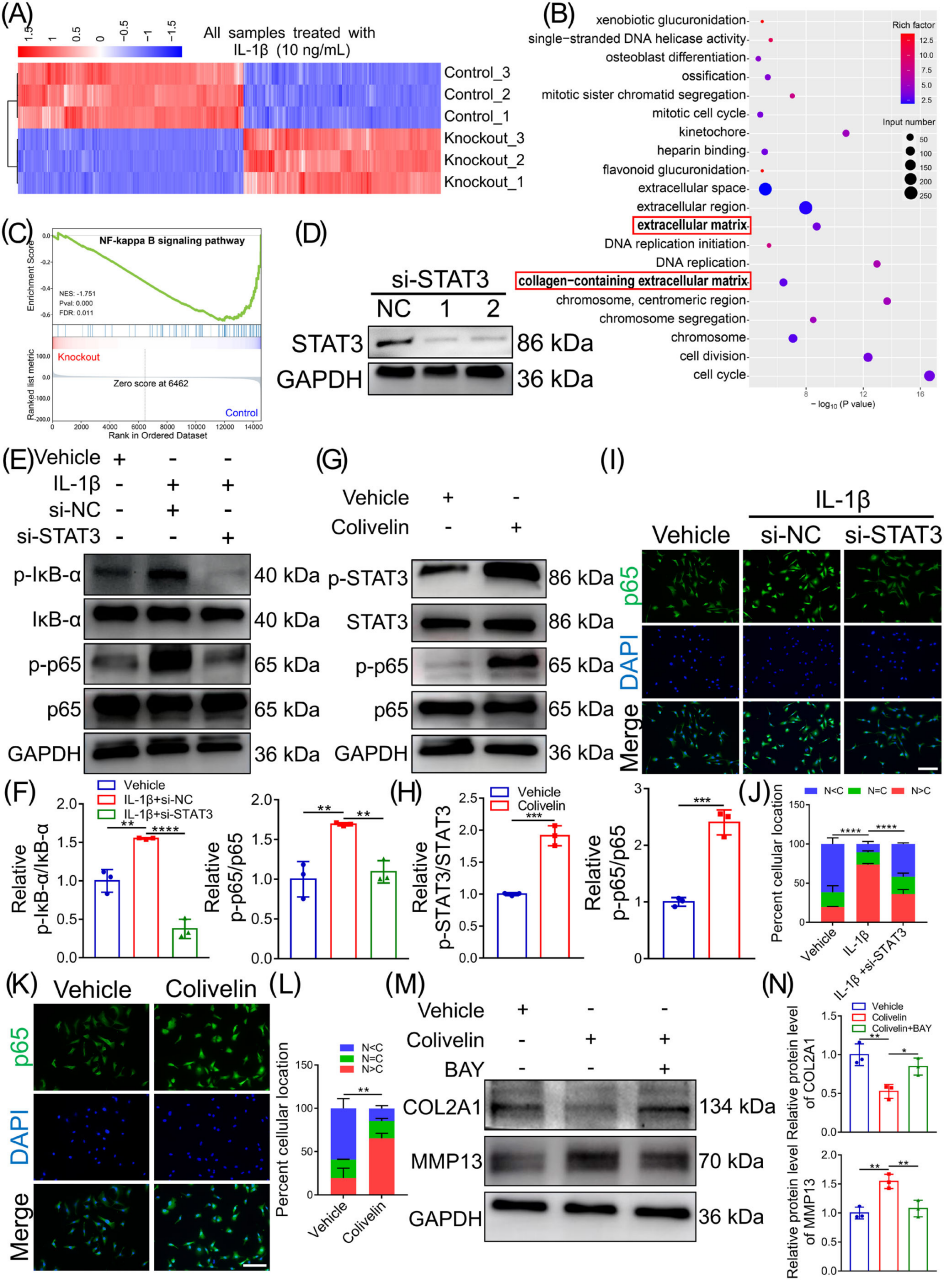
Phosphorylated signal transducer and activator of transcription 3 (STAT3) regulates chondrocyte matrix metabolism by activating the NF‐κB pathway. (A) Heatmap of RNA‐seq data showing all differentially expressed genes (DEGs) in STAT3 knockout groups versus control groups, both treated with IL‐1β (10 ng/mL). (B) Gene ontology (GO) enrichment analysis of the biological process category. The red box highlights extracellular matrix (ECM)‐related processes. (C) GSEA showing enrichment of the NF‐κB pathway in RNA‐seq data. (D) Immunoblotting to validate STAT3 knockout efficiency. (E, F) Immunoblotting analysis of p‐IκB‐α, IκB‐α, p‐p65 and p65 in IL‐1β‐treated (10 ng/mL) chondrocytes transfected with si‐NC or si‐STAT3. The band intensities were quantified and normalised against GAPDH. (G, H) Immunoblotting analysis of p‐STAT3, STAT3, p‐p65 and p65 in chondrocytes treated with vehicle or Colivelin (.5 µM). The band intensities were quantified and normalised against GAPDH. (I–L) Representative immunofluorescence (IF) images and quantitative analyses of the nuclear translocation of p65 (green) in chondrocytes grouped as shown in the figure. DAPI (blue) stains nuclei. N < C, cytoplasmic p65 exceeds nuclear p65. N = C, comparable nuclear and cytoplasmic p65. N > C, nuclear p65 predominates over cytoplasmic p65. ^*^Statistical evaluation focused on comparing the proportion of N > C cells among the groups. Scale bars: 50 µm. (M, N) Immunoblotting analysis of COL2A1 and MMP13 in chondrocytes treated with Colivelin and/or BAY (1 µM). BAY, BAY 11–7085. The band intensities were quantified and normalised against GAPDH. Quantitative analyses were based on three biological replicates. ^*^ *p* < .05. ^**^ *p* < .01. ^***^ *p* < .001. ^****^ *p* < .0001.

To elucidate the mechanism underlying p‐STAT3 modulates ECM metabolism, we performed RNA‐seq on IL‐1β‐treated chondrocytes with or without STAT3 knockout. A heatmap of DEGs clearly distinguished the transcriptional profiles of the two groups (Figure 4A). GO enrichment analysis in biological process categories identified significant enrichment of ECM‐related terms such as ECM and collagen‐containing ECM, further confirming STAT3's role in chondrocyte matrix homeostasis (Figure 4B). Notably, Gene set enrichment analysis (GSEA) indicated that the NF‐κB pathway (a key regulator of inflammation and matrix degradation) was significantly downregulated upon STAT3 knockout (Figure 4C).

To establish a causal link between p‐STAT3 and NF‐κB activation, si‐STAT3 was constructed and its knockdown efficiency validated by immunoblotting (Figure 4D). STAT3 knockdown reversed IL‐1β‐induced phosphorylation of IκB‐α and p65, the key components of the NF‐κB pathway, without altering their total protein levels (Figure 4E,F). Conversely, Colivelin‐induced STAT3 phosphorylation increased p‐p65 levels, confirming that p‐STAT3 promotes NF‐κB activation (Figure 4G,H). IF staining corroborated these findings: STAT3 knockdown blocked IL‐1β‐triggered p65 nuclear translocation, while Colivelin robustly induced p65 nuclear import (Figure 4I–L).

To confirm that NF‐κB activation mediates p‐STAT3‐induced ECM dysfunction, we treated chondrocytes with Colivelin alone or in combination with the selective NF‐κB inhibitor BAY 11‐7085. As shown by immunoblotting, BAY 11‐7085 treatment reversed Colivelin‐induced COL2A1 downregulation and MMP13 upregulation (Figure 4M,N). Furthermore, RT‐qPCR and ELISA analyses showed that BAY 11‐7085 also reversed Colivelin‐induced upregulation of additional NF‐κB target genes, and the elevated secretion of IL‐1β, IL‐6 and TNF‐α (Figure S5A,B). These results indicate that p‐STAT3 regulates chondrocyte ECM metabolism and inflammatory responses by activating the NF‐κB pathway.

### 3.5 Phosphorylated STAT3 directly binds the DCLK1 promoter to upregulate its expression

**FIGURE 5:**
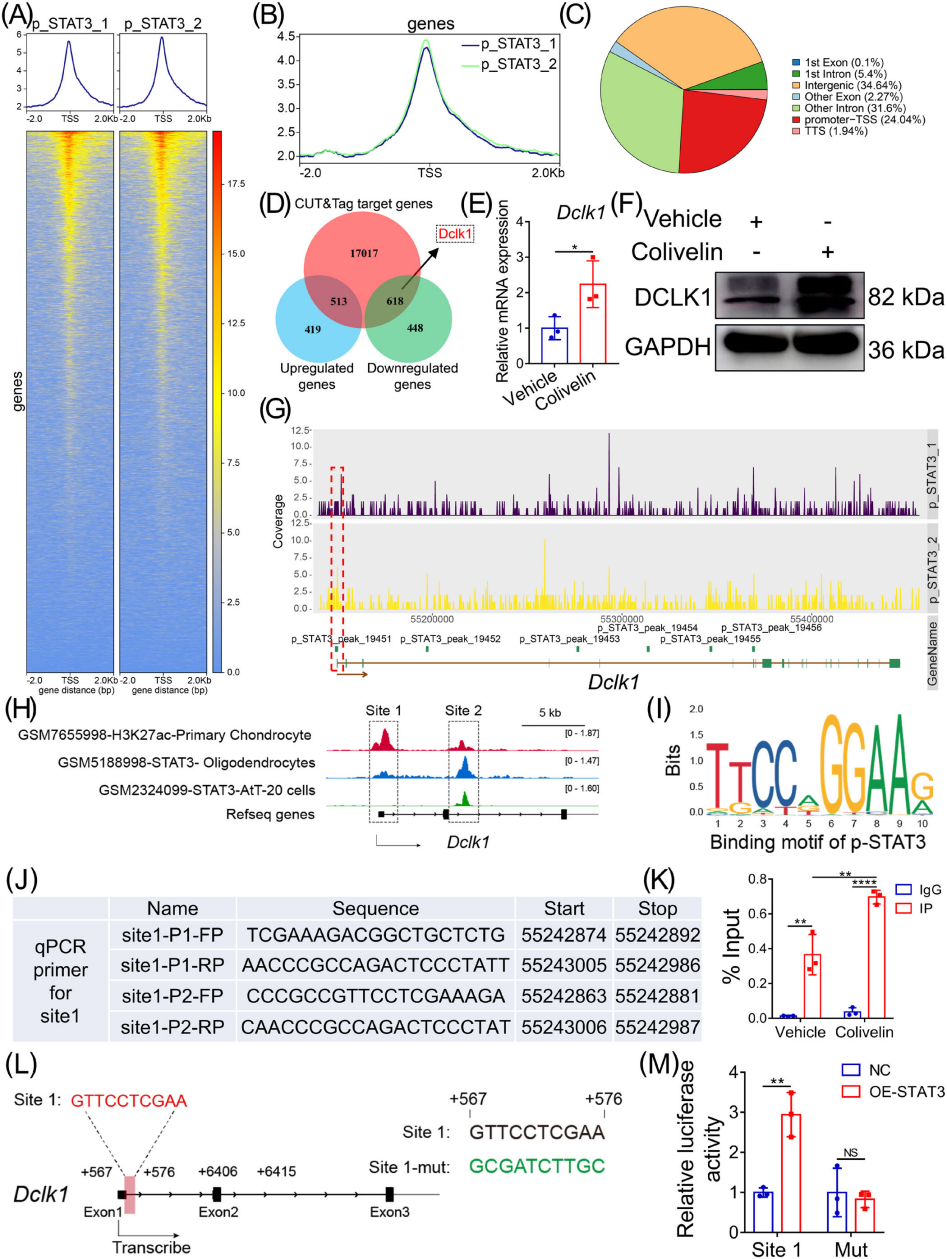
Phosphorylated signal transducer and activator of transcription 3 (STAT3) directly binds the DCLK1 promoter to upregulate its expression. (A) Heatmap of p‐STAT3 by cleavage under targets and tagmentation sequencing (CUT&Tag‐seq) technology (*n* = 2 biological replicates). (B) The distribution of the reads around the transcription start sites (TSSs) of target genes. (C) Pie chart illustrating the genomic distribution of p‐STAT3‐binding peaks. (D) Venn diagram of overlapping genes between differentially expressed genes (DEGs) from RNA‐seq and p‐STAT3 target genes from CUT&Tag. (E, F) quantitative real‐time PCR (RT‐qPCR) (E) and immunoblotting (F) of doublecortin‐like kinase 1 (DCLK1) in chondrocytes treated with vehicle or Colivelin (.5 µM). (G) p‐STAT3 peak enrichment in the DCLK1 promoter region by CUT&Tag analysis. (H) IGV tracks of STAT3 ChIP‐seq (GSM5188998, human Th17 cells; GSM2324099, mouse T cells) and H3K27ac ChIP‐seq signals (GSM7655998, mouse chondrocytes) data downloaded from GEO (https://www.ncbi.nlm.nih.gov/geo/), showing enrichment at the *Dclk1* locus. Two putative STAT3‐binding sites (Site 1 and Site 2) were indicated. (I) Schematic of Site 1 with its DNA sequence containing a classic STAT3‐binding motif. (J, K) Based on the peak sequence at Site 1, two primer pairs were designed (J) and ChIP‐qPCR results (K) showing p‐STAT3 binding to the DCLK1 promoter (*n* = 3 biological replicates). (L) Sequence of wild‐type Site 1 and the mutant Site 1. (M) Dual‐luciferase assay showing luciferase activity driven by wild‐type or mutant DCLK1 promoter (Site 1) in chondrocytes transfected with OE‐STAT3 or NC. Quantitative analyses were based on three biological replicates. NS, not significant. ^*^ *p* < .05. ^**^ *p* < .01. ^****^ *p* < .0001.

Given that p‐STAT3 functions as a transcription factor, we combined RNA‐seq and CUT&Tag‐seq to identify its downstream targets in chondrocytes. Application of CUT&Tag‐seq to map the genome‐wide binding sites of p‐STAT3 in chondrocytes revealed distinct p‐STAT3‐binding profiles across biological replicates (Figure 5A), with binding enriched near transcription start sites (TSSs; Figure 5B) and a large proportion of peaks localised to promoter regions (Figure 5C). Integrative analysis identified 513 upregulated and 618 downregulated DEGs that overlapped with p‐STAT3 target genes (Figure 5D). Candidate p‐STAT3 target genes were prioritised by expression fold change, promoter peak enrichment and OA‐relevant functions, with top‐ranked candidates listed in Table S5. Given that DCLK1 activates the NF‐κB pathway^29^ and its expression was upregulated by STAT3 activation (Figure 5E,F), we selected DCLK1 for mechanistic validation.

CUT&Tag analysis showed a robust p‐STAT3‐binding peak in the DCLK1 promoter (Figure 5G). Integration with public ChIP‐seq datasets (GSM5188998, GSM2324099 for STAT3; GSM7655998 for H3K27ac) identified two putative p‐STAT3‐binding sites (Site 1 and Site 2), with Site 1 containing a canonical STAT3‐binding motif (GTTCCTCGAA; Figure 5H,I). To corroborate these findings, two primer pairs targeting the Site 1 peak (Figure 5J) were used in ChIP‐qPCR to assess p‐STAT3 binding to the DCLK1 promoter in chondrocytes. The anti‐p‐STAT3 antibody, but not IgG, precipitated the promoter region containing Site 1, and Colivelin treatment enhanced this binding (Figure 5K). Additionally, the putative WT and mutant binding sequences for Site 1 were designed based on the motif and JASPAR database (Figure 5L). Dual‐luciferase experiments in chondrocytes showed that STAT3 overexpression (OE‐STAT3) strongly activated the WT DCLK1 promoter, while the mutant promoter abolished this effect (Figure 5M). Overall, these results confirm that p‐STAT3 directly binds Site 1 in the DCLK1 promoter to activate its transcription.

### 3.6 DCLK1 activates the NF‐κB pathway by interacting with IKKβ and promoting its phosphorylation

**FIGURE 6:**
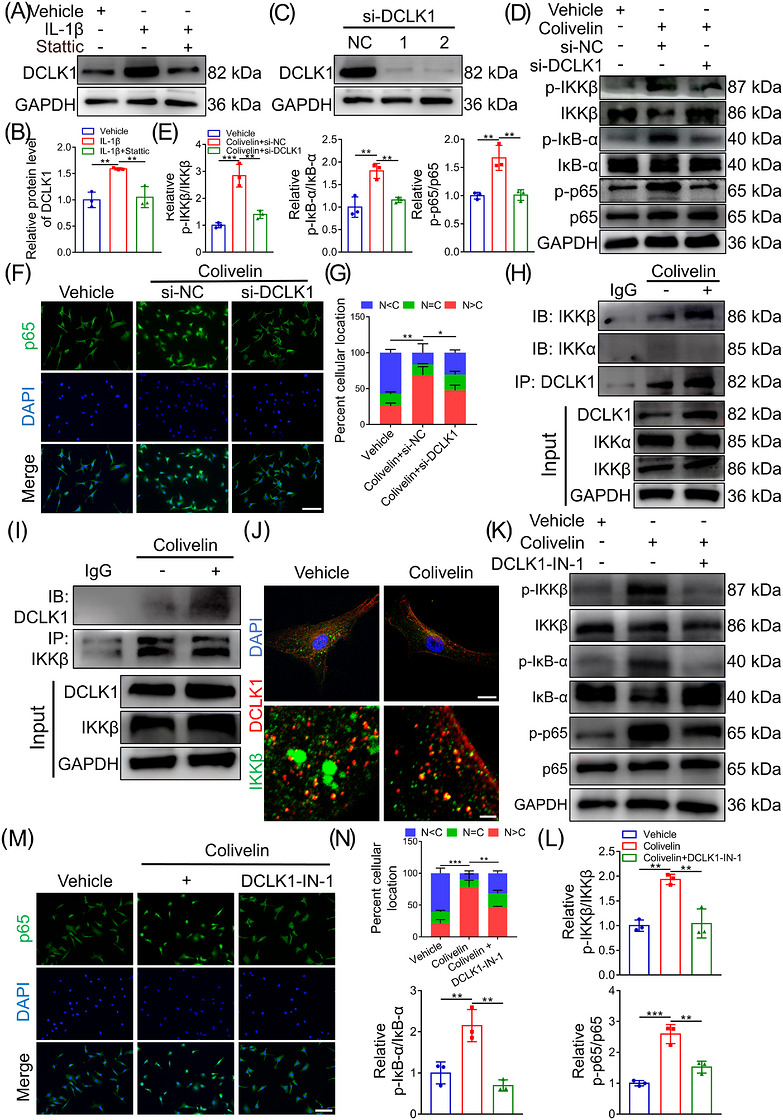
Doublecortin‐like kinase 1 (DCLK1) activates the NF‐κB pathway by interacting with IKKβ and promoting its phosphorylation. (A, B) Immunoblotting analysis of DCLK1 in chondrocytes treated with IL‐1β (10 ng/mL) and/or Stattic (1 µM). The band intensities were quantified and normalised against GAPDH. (C) Immunoblotting to validate DCLK1 knockout efficiency. (D, E) Immunoblotting analysis of p‐IκB‐α, IκB‐α, p‐p65 and p65 in chondrocytes transfected with si‐NC or si‐DCLK1 and treated with Colivelin (.5 µM). The band intensities were quantified and normalised against GAPDH. (F, G) Representative immunofluorescence (IF) images and quantification of p65 nuclear translocation (green) in chondrocytes. DAPI (blue) stains nuclei. N < C, cytoplasmic p65 exceeds nuclear p65. N = C, comparable nuclear and cytoplasmic p65. N > C, nuclear p65 predominates over cytoplasmic p65. ^*^Statistical evaluation focused on comparing the proportion of N > C cells among the groups. Scale bars: 50 µm. (H, I) Co‐immunoprecipitation (Co‐IP) results showing the interaction between DCLK1 and IKKβ (but not IKKα) in chondrocytes with or without Colivelin (.5 µM). (J) Confocal IF images depicting the colocalisation of DCLK1 (red) and IKKβ (green) in the cytoplasm of chondrocytes. DAPI (blue) stains nuclei. Scale bars: 20 µm (upper), 2 µm (lower). (K, L) Immunoblotting analysis of p‐IKKβ, IKKβ, p‐IκB‐α, IκB‐α, p‐p65 and p65 in chondrocytes treated with Colivelin (.5 µM) and/or DCLK1‐IN‐1 (1 µM). The band intensities were quantified and normalised against GAPDH. (M, N) Representative IF images and quantitative analyses of p65 (green) in chondrocytes. DAPI (blue) stains nuclei. Scale bars: 50 µm. Quantitative analyses were based on three biological replicates. ^*^ *p* < .05. ^**^ *p* < .01. ^***^ *p* < .001.

To clarify the role of DCLK1 in p‐STAT3‐regulated NF‐κB activation, immunoblotting confirmed that IL‐1β treatment significantly increased DCLK1 protein levels, an effect reversed by the STAT3 inhibitor Stattic, indicating a positive regulatory link between p‐STAT3 and DCLK1 (Figure 6A,B). We then employed siRNA‐mediated DCLK1 knockdown (Figure 6C) and found that DCLK1 depletion blocked Colivelin‐induced phosphorylation of IκB‐α and p65 (Figure 6D,E), as well as p65 nuclear translocation (Figure 6F,G). To further confirm the specificity of DCLK1‐mediated effects, we generated DCLK1 knockout chondrocytes using a CRISPR/Cas9 lentiviral system targeting Dclk1 (Figure S6A). In DCLK1 knockout chondrocytes, Colivelin‐induced phosphorylation of IKKβ, IκB‐α and p65 was completely abolished (Figure S6B), and the downstream ECM dysregulation was fully reversed (Figure S6C). These results demonstrate that DCLK1 as an essential downstream effector of p‐STAT3 in NF‐κB activation.

DCLK1 contains an N‐terminal doublecortin (DCX) domain and a C‐terminal serine/threonine kinase domain, the latter mediating signalling through phosphorylation of downstream targets.^31^ Given that NF‐κB is activated by a phosphorylation cascade, the interaction between DCLK1 and IκB kinase (IKK) complex (IKKα and IKKβ) was examined. Co‐immunoprecipitation (Co‐IP) revealed a specific interaction between DCLK1 and IKKβ (but not IKKα) in chondrocytes, which was enhanced by STAT3 activation (Figure 6H,I). Confocal IF staining further confirmed their cytoplasmic colocalisation in situ (Figure 6J). Given DCLK1 kinase activity, we tested whether its interaction with IKKβ promotes IKKβ phosphorylation. Colivelin treatment increased phosphorylation of IKKβ, IκB‐α and p65, while DCLK1‐IN‐1, DCLK1 kinase inhibitor, reversed these effects (Figure 6K,L) without altering DCLK1 protein levels (Figure S7A,B). IF staining further confirmed that DCLK1‐IN‐1 blocked Colivelin‐induced p65 nuclear translocation (Figure 6M,N). These results indicate that DCLK1 activates the NF‐κB pathway by interacting with IKKβ and promoting its phosphorylation. Moreover, DCLK1‐IN‐1 reversed Colivelin‐induced ECM dysregulation in chondrocytes (Figure S7C,D), highlighting DCLK1 as a potential therapeutic target.

### 3.7 DCLK1 is an essential downstream target of STAT3 in OA, and its inhibition has therapeutic potential

**FIGURE 7:**
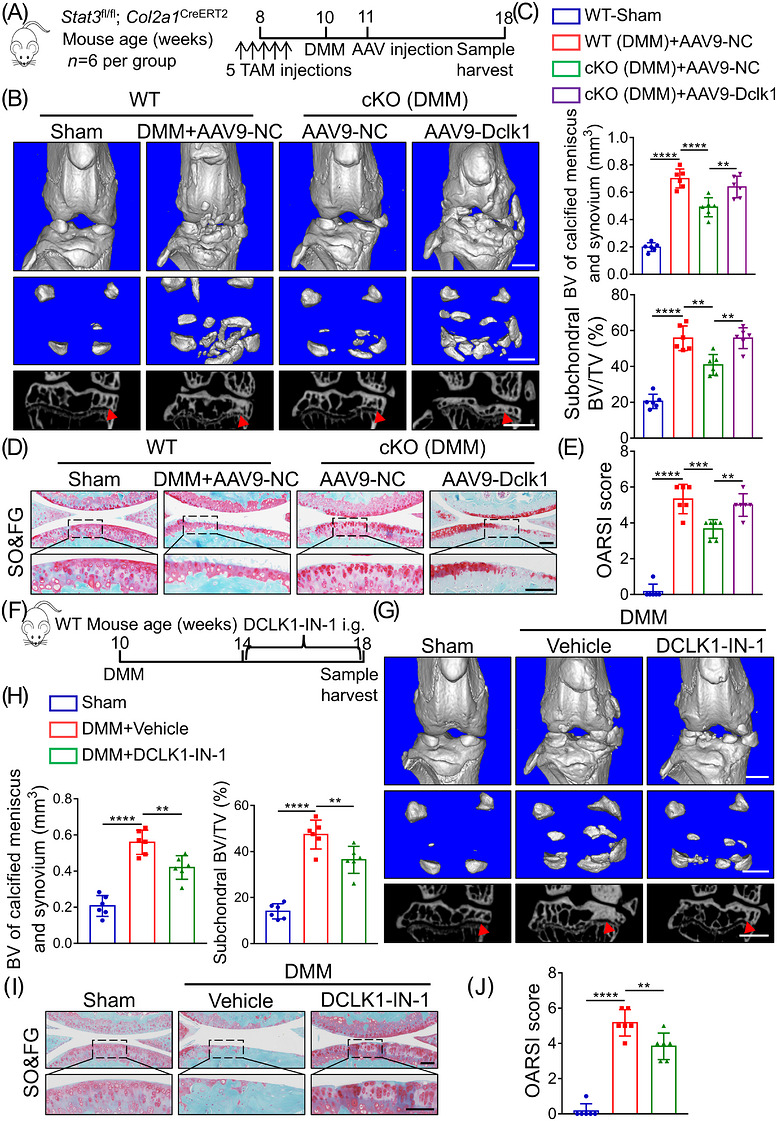
Doublecortin‐like kinase 1 (DCLK1) is a key downstream target of signal transducer and activator of transcription 3 (STAT3) in osteoarthritis (OA), and its inhibition alleviates OA. (A) Schematic overview of the experimental protocol. *Stat3* ^fl/fl^; *Col2a1* ^CreERT2^ mice aged 8 months were administered intraperitoneal TAM (cKO, *n* = 6) or corn oil (WT, *n* = 6) for 5 consecutive days. Two weeks later, mice in the cKO and wild‐type (WT) groups underwent DMM or sham surgery respectively. One week after surgery, WT and cKO mice received intra‐articular injection of AAV9‐Dclk1 (5 × 10^9^ vg in 10 µL) or AAV9‐NC. Knee joints were obtained at 18 weeks of age. TAM, tamoxifen. DMM, destabilisation of the medial meniscus. (B) Three‐dimensional reconstruction from micro‐CT scans of knee joints. Red arrows, medial tibial subchondral bone. Scale bar: 1.0 mm. (C) The calcified meniscus and synovial tissue volume, and BV/TV of the medial tibial subchondral bone, were analysed by micro‐CT. BV, bone volume. TV, tissue volume. (D, E) Safranin O & Fast Green (SO&FG) staining of knee joint sections and quantification of Osteoarthritis Research Society International (OARSI) score. Scale bar: 100 µm. (F) Schematic overview of the experimental protocol. Ten‐week‐old WT mice underwent DMM or sham surgery. Four weeks post‐surgery, DCLK1‐IN‐1 was administered intragastrically at 10 mg/kg once daily for 4 weeks. The control group was administered vehicle (.5% DMSO in saline). Knee joints were obtained at 18 weeks of age (*n* = 6 per group). i.g., intragastrically. TAM, tamoxifen. (G, H) Three‐dimensional reconstruction and quantitative analysis from micro‐CT scans. (I, G) SO&FG staining of knee joint sections and quantification of OARSI score. NS, not significant. ^*^ *p* < .05. ^**^ *p* < .01. ^***^ *p* < .001. ^****^ *p* < .0001.

To confirm DCLK1 as a functional downstream effector of STAT3 in vivo, AAV9‐Dclk1 was injected intra‐articularly into STAT3 cKO mice after DMM surgery, as outlined in Figure 7A. As expected, STAT3 deficiency significantly reduced DMM‐induced calcification of meniscus and synovium, as well as subchondral bone BV/TV. These protective effects were completely abrogated by DCLK1 overexpression (Figure 7B,C). Moreover, histological analysis showed that DCLK1 overexpression reversed the attenuation of cartilage matrix loss and decreased OARSI scores in cKO‐DMM mice (Figure 7D,E). Consistently, DCLK1 overexpression disrupted ECM homeostasis (Figure S8A,B) and restored p‐p65 expression without altering p‐STAT3 levels (Figure S9A,B), unequivocally establishing DCLK1 as the critical downstream target of p‐STAT3 that activates NF‐κB signalling and drives OA pathogenesis.

To assess the therapeutic relevance of DCLK1 inhibition, C57BL/6 mice were treated as depicted in Figure 7F. At 8 weeks post‐DMM, DCLK1‐IN‐1 treatment markedly reduced joint destruction, calcified meniscus and synovial tissue volumes, and medial tibial subchondral bone BV/TV (Figure 7G,H). Cartilage erosion was attenuated, with lower OARSI scores and improved matrix preservation (Figure 7I,J). Consistent with these structural improvements, IHC confirmed that DCLK1‐IN‐1 restored ECM homeostasis by upregulating COL2A1 and downregulating MMP13 (Figure S8C,D). In addition, DCLK1‐IN‐1 treatment significantly reduced the p‐p65 expression in articular cartilage without affecting p‐STAT3 levels (Figure S9C,D), further validating the p‐STAT3/DCLK1/NF‐κB axis in vivo. Taken together, our findings suggest that DCLK1 inhibition mitigates experimental OA, underscoring its potential as a therapeutic target.

## 4 DISCUSSION

Here, we first generated chondrocyte‐specific STAT3 knockout mice and uncovered a novel p‐STAT3/DCLK1/IKKβ/NF‐κB axis that regulates chondrocyte ECM homeostasis. STAT3 ablation attenuated both spontaneous age‐related and DMM‐induced post‐traumatic OA, with increased COL2A1 and decreased MMP13 expression. Mechanistically, p‐STAT3 directly bound a canonical motif in the DCLK1 promoter to activate its transcription. Upregulated DCLK1 physically interacted with and phosphorylated IKKβ, triggering NF‐κB activation and subsequent ECM dysregulation. Critically, pharmacological inhibition of DCLK1 with DCLK1‐IN‐1 markedly attenuated OA pathology in the DMM model, supporting the p‐STAT3/DCLK1 axis as an attractive therapeutic candidate for OA.

Our findings advance the understanding of STAT3 signalling in OA pathogenesis. We previously reported that RORα drives OA progression by regulating the IL‐6/STAT3 pathway, specifically through interacting with STAT3 and binding to the IL‐6 promoter to promote STAT3 phosphorylation and subsequent inflammatory responses.^32^ Here, we extend these findings by delineating the downstream effector mechanism through which p‐STAT3 exerts its catabolic effects in chondrocytes. While reciprocal transcriptional crosstalk between STAT3 and NF‐κB has been documented in other inflammatory contexts,^33^, ^34^, ^35^ whether p‐STAT3 directly activates upstream components of the NF‐κB cascade in OA chondrocytes remained unknown. We identify DCLK1 as an essential intermediary through which STAT3 triggers NF‐κB‐mediated cartilage destruction.

To unequivocally identify direct transcriptional targets of p‐STAT3, we employed CUT&Tag‐seq, a sensitive method well‐suited to limited primary chondrocytes.^36^ Integrated CUT&Tag‐seq and RNA‐seq analyses pinpointed DCLK1. Among the validated targets, DCLK1 has been extensively characterised in cancer and neurobiology, where it regulates microtubule dynamics, cell migration and stemness.^26^, ^37^, ^38^ Recent studies have established a growing link between DCLK1 and inflammatory diseases. For instance, DCLK1 deficiency attenuates obesity‐induced cardiomyopathy by suppressing RIP2/TAK1 and macrophage inflammation.^39^ Furthermore, studies in human lung epithelial cells demonstrated that DCLK1 activates NF‐κB pathway to induce expression of the inflammatory cytokine IL‐8/CXCL8.^40^ Given these findings, exploring the role of DCLK1 in the inflammatory pathology of OA is warranted. Our study demonstrates that in chondrocytes, DCLK1 specifically interacts with IKKβ (but not IKKα), an interaction enhanced by p‐STAT3 activation, thus extending the DCLK1‐IKKβ‐NF‐κB axis to cartilage biology. This suggests a conserved role for DCLK1‐IKKβ interaction in inflammatory signalling across cell types and diseases.^28^ Moreover, we identified that p‐STAT3 directly transactivates DCLK1, functionally linking this master inflammatory transcription factor to the NF‐κB pathway in joint degeneration.

Notably, the regulatory hierarchy between STAT3 and DCLK1 appears to be context‐dependent. In cancer, prevailing evidence supports a paradigm in which DCLK1 functions upstream of STAT3: DCLK1 inhibition suppresses STAT3 phosphorylation in colorectal cancer,^41^ DCLK1 activates JAK1/STAT3 signalling via IL‐6 upregulation in triple‐negative breast cancer,^42^ and ELF1‐driven DCLK1 transcription upregulates JAK2/STAT3 in pancreatic cancer.^43^ In contrast, our study reveals a reversed hierarchy in OA chondrocytes, where p‐STAT3 acts upstream of DCLK1 by directly binding the DCLK1 promoter to activate its transcription, and DCLK1 knockdown does not affect p‐STAT3 levels (Figure S10), confirming unidirectional regulation. This divergence likely reflects distinct cellular contexts: in cancer, DCLK1 serves as a proximal driver sustaining STAT3‐mediated stemness, whereas in the inflammatory milieu of OA, STAT3 functions as a master transcriptional regulator that deploys DCLK1 as a downstream effector to link inflammatory signals to NF‐κB‐driven matrix degradation. These findings highlight the importance of tissue‐ and disease‐specific considerations when therapeutically targeting the STAT3–DCLK1 axis.

The translational potential of targeting p‐STAT3/DCLK1 axis is underscored by our intervention studies. Forced overexpression of DCLK1 completely abrogates the protective phenotype in cKO mice confirms its role as a critical downstream effector of STAT3 in vivo. More importantly, pharmacological inhibition of DCLK1 with DCLK1‐IN‐1 significantly attenuated OA progression, highlighting its promise as a therapeutic target, and consistent with the efficacy of DCLK1 blockade in other inflammatory diseases.^44^, ^45^, ^46^ Targeting downstream kinases such as DCLK1 offers a distinct therapeutic advantage over inhibiting upstream master regulators like STAT3: it selectively mitigates NF‐κB‐driven cartilage catabolism while avoiding the broad cellular dysregulation and systemic toxicity associated with global STAT3 inhibition.

This research has some limitations. First, we focused exclusively on chondrocytes, but OA is a complex multi‐tissue disease involving synovial fibroblasts, subchondral osteoblasts and macrophages.^47^ Future studies should examine whether the p‐STAT3/DCLK1 axis operates in other joint tissues to contribute to OA pathogenesis. Second, the small human sample size limits the ability to correlate p‐STAT3/DCLK1 expression with OA severity and evaluate its biomarker potential. Finally, the long‐term biosafety profile of the DCLK1 inhibitor DCLK1‐IN‐1 remains incompletely characterised. Further optimisation of its bioavailability, joint tissue permeability and specificity is required to advance its potential for clinical application.

## 5 CONCLUSIONS

**FIGURE 8:**
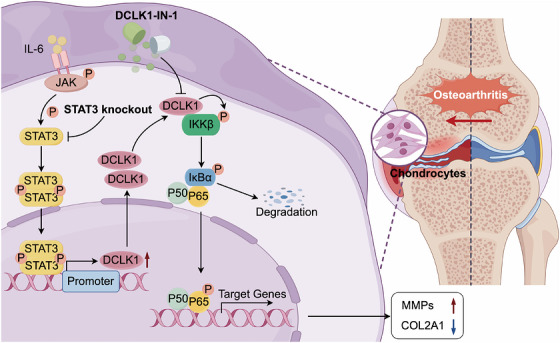
Schematic of the p‐STAT3/DCLK1/NF‐κB axis in osteoarthritis (OA) pathogenesis. In OA chondrocytes, inflammatory cytokines trigger signal transducer and activator of transcription 3 (STAT3) phosphorylation and nuclear translocation, where it binds the doublecortin‐like kinase 1 (DCLK1) promoter to activate transcription. Upregulated DCLK1 interacts with and phosphorylates IKKβ, activating the NF‐κB cascade. This drives MMP13 upregulation and COL2A1 downregulation, promoting cartilage degeneration. Genetic STAT3 ablation or pharmacological DCLK1 inhibition disrupts this axis, restoring extracellular matrix (ECM) homeostasis and attenuating OA.

In summary, our study identify the p‐STAT3/DCLK1/IKKβ/NF‐κB axis as a critical mechanism in OA pathogenesis and highlight DCLK1 as a promising therapeutic target for OA treatment (Figure 8).

## AUTHOR CONTRIBUTIONS

All the authors have critically reviewed and approved the final manuscript to be published. Conception and design of study: Bo Gao, Yilin Liu, Wenjie Gao and Yuqiang Wang. Acquisition of data: Pengfei Li, Chipiu Wong, Yuqiang Wang and Yichen Que. Analysis and/or interpretation of data: Chipiu Wong and Yuqiang Wang. Drafting the manuscript: Pengfei Li and Chipiu Wong. Revising the manuscript critically for important intellectual content: Bo Gao and Tongzhou Liang.

## CONFLICT OF INTEREST STATEMENT

The authors declare no conflicts of interest.

## ETHICS STATEMENT

The research protocol has been approved by the Clinical Trials Ethics Committee of the First Affiliated Hospital of Zhengzhou University (Approval No.: 2025‐KY‐0632‐001). The animal experiments were approved by the Institutional Animal Care and Use Committee of Sun Yat‐sen University (Approval No.: SYSU‐IACUC‐2022‐001293).

## CONSENT FOR PUBLICATION

All the authors reviewed the final version of the manuscript and approved it for publication.

## Supporting information



Supporting Information

## Data Availability

The datasets generated and analysed during the current study are available from the corresponding author upon reasonable request.
