# Supplementary material for: DCLK1‐dependent NF‐κB activation mediates p‐STAT3‐induced osteoarthritis progression
Source: Clin Transl Med. 2026 Jun 15;16(6):e70712. doi: 10.1002/ctm2.70712 (PMC13269830; doi:10.1002/ctm2.70712)
Supplement: Supplementary file 1 — Supporting Information [file CTM2-16-e70712-s001.docx]

**Supplementary Materials**

**List of Materials**

Supplementary Methods

Fig. S1.

Fig. S2.

Fig. S3.

Fig. S4.

Fig. S5.

Fig. S6.

Fig. S7.

Fig. S8.

Fig. S9.

Fig. S10.

Table S1.

Table S2.

Table S3.

Table S4.

Table S5.

**Supplementary Methods**

**Inclusion and exclusion criteria for clinical human samples**

Inclusion criteria for osteoarthritis (OA) group

1. Radiographically confirmed primary knee osteoarthritis with a Kellgren–Lawrence grade ≥ III;
2. Scheduled for total knee arthroplasty (TKA) with intraoperative availability of tibial plateau cartilage and synovial tissue;
3. Age ≥ 50 years.

Exclusion criteria for OA group

1. Presence of other joint diseases, including rheumatoid arthritis, gouty arthritis, post-traumatic arthritis, or infectious arthritis;
2. Severe systemic diseases, such as uncontrolled diabetes, malignant tumors, immunodeficiency disorders, etc;
3. Use of glucocorticoids or immunosuppressive agents

Control group

Normal controls with no history of arthritis or chronic joint pain/dysfunction,

The normal group consisted of patients without a history of arthritis or chronic joint pain/dysfunction, who underwent TKA due to joint trauma.

**Antibodies**

The following primary antibodies were used: anti-STAT3 (CST 9139, 1:1,000), anti- p-STAT3 (CST 9145, 1: 1,000), anti-COL2A1 (Proteintech 28459-1-AP, 1:1,000), anti-MMP13 (Abcam 39012, 1:1,000), anti-IκB-α (CST 4814T, 1:1,000), anti-phospho-IκB-α (CST 2859T, 1:1,000), anti-NF-kB p65 (Proteintech 10745-1-AP, 1:1,000), anti-phospho-NF-kB p65 (Proteintech 82335, 1:1,000), anti-IKKα (Proteintech 84372-4-RR, 1:1,000), anti-IKKβ (Proteintech 15649-1-AP, 1:1,000), anti-phospho-IKKβ (CST 2697, 1:1,000), anti-DCLK1 (Proteintech 21699-1-AP, 1:1,000), and anti-GAPDH (Proteintech 60004-1-Ig, 1:10,000). Secondary antibodies were goat anti-rabbit IgG (Servicebio GB23303, 1:10,000) and goat anti-mouse IgG (Servicebio GB23301, 1:10,000).

**Supplementary Figures**

**
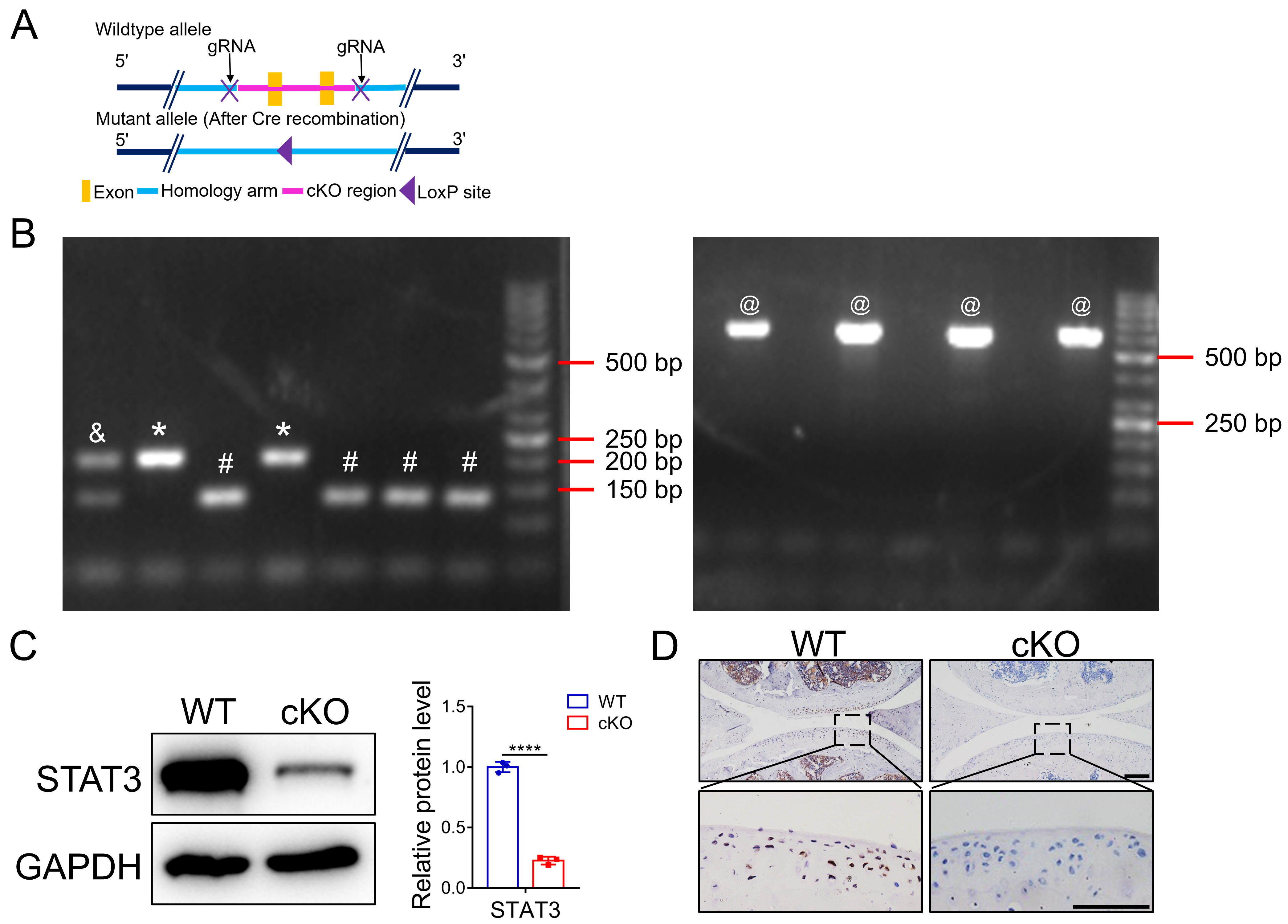
**

**Fig. S1. Validation of chondrocyte-specific STAT3 knockout (cKO) mice.**

(A) Schematic of the Stat3 floxed allele and Cre-mediated recombination. (B) Genotype identification by gel electrophoresis. The band labeled “&” represents the heterozygous genotype; “*” represents the homozygous mutant genotype; “#” represents the wild-type genotype; “@” represents the Col2a1-CreERT2 genotype. (C) Immunoblotting and quantification of STAT3 protein levels in articular cartilage from WT and cKO mice, with GAPDH as an internal reference control (n = 3 biological replicates). (D) IHC staining to verify STAT3 knockout efficiency. Scale bar: 100 μm. ^****^*P* < 0.0001.


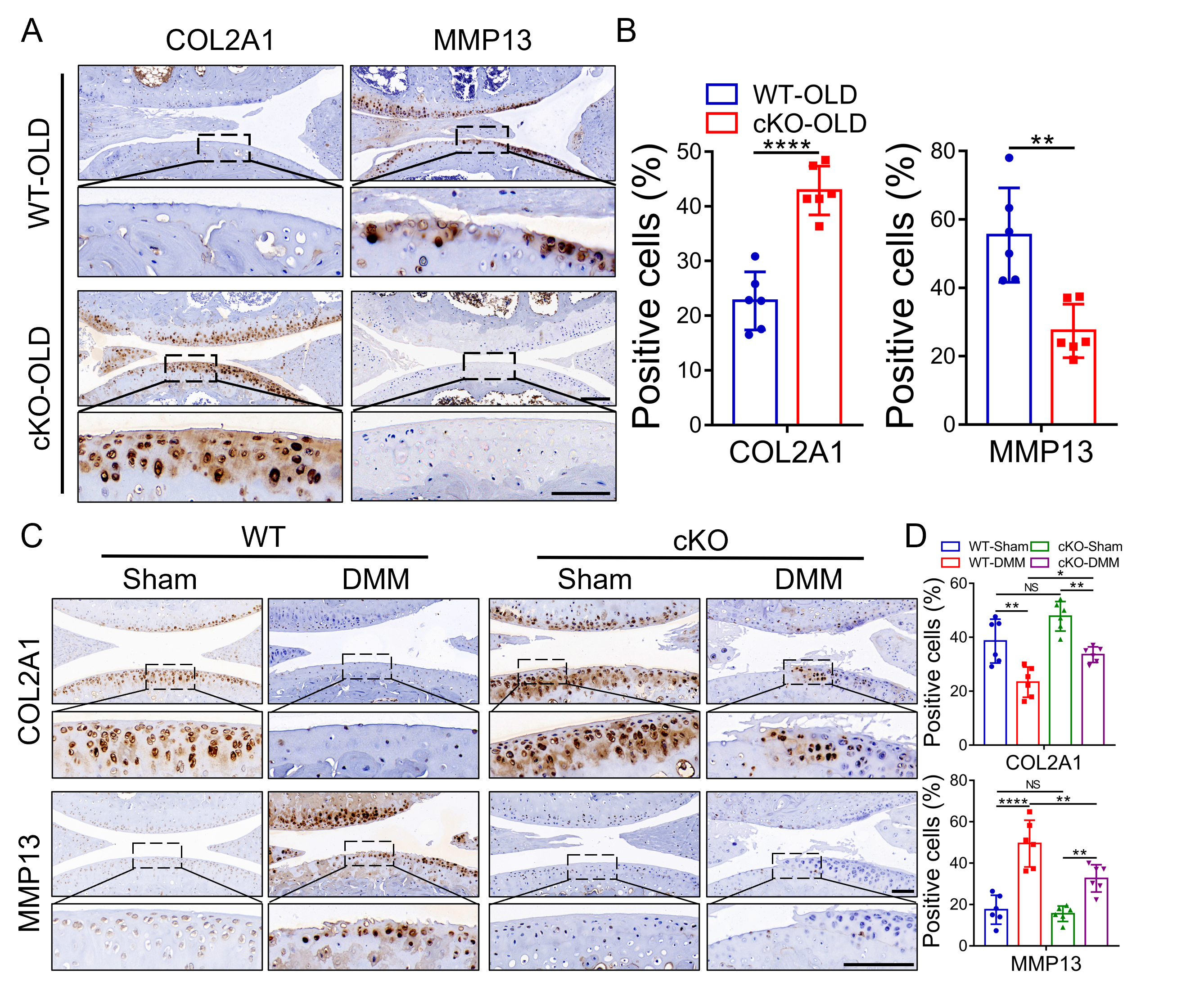


**Fig. S2. Chondrocyte-specific STAT3 deletion restores ECM homeostasis in spontaneous and DMM-induced OA mice.**

(A, C) IHC staining of knee joint sections showing COL2A1 and MMP13 expression in articular cartilage. Scale bar: 100 μm. (B, D) Quantification of COL2A1 and MMP13 expression. Quantitative analyses were based on six biological replicates. NS, not significant. ^*^*P* < 0.05. ^**^*P* < 0.01. ^****^*P* < 0.0001.


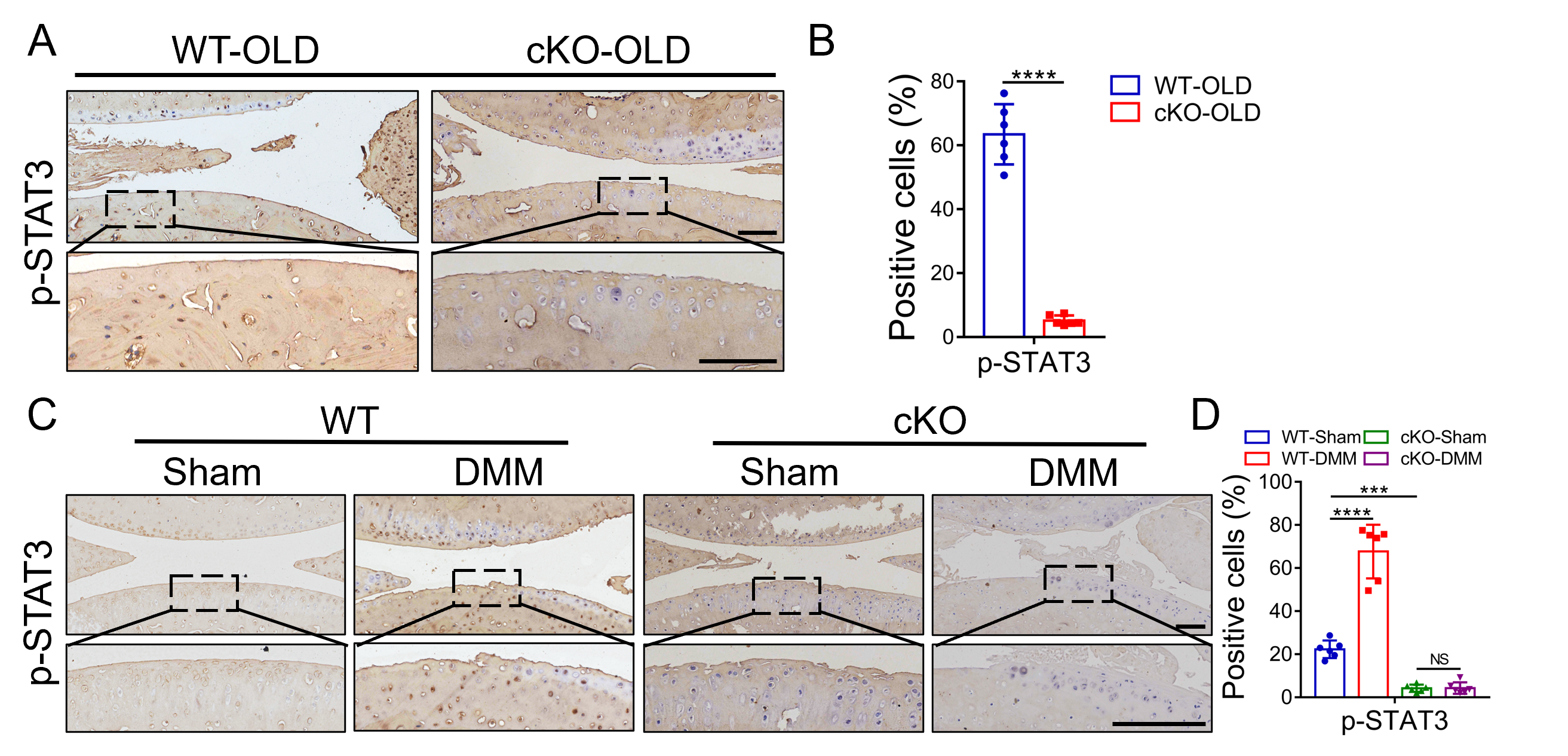


**Fig. S3. IHC analysis of p-STAT3 in spontaneous aging and DMM-induced osteoarthritis mouse models.**

(A, C) IHC staining of knee joint sections showing p-STAT3 expression in articular cartilage. Scale bar: 100 μm. (B, D) Quantification of p-STAT3 expression. Quantitative analyses were based on six biological replicates. NS, not significant. ^***^*P* < 0.001. ^****^*P* < 0.0001.


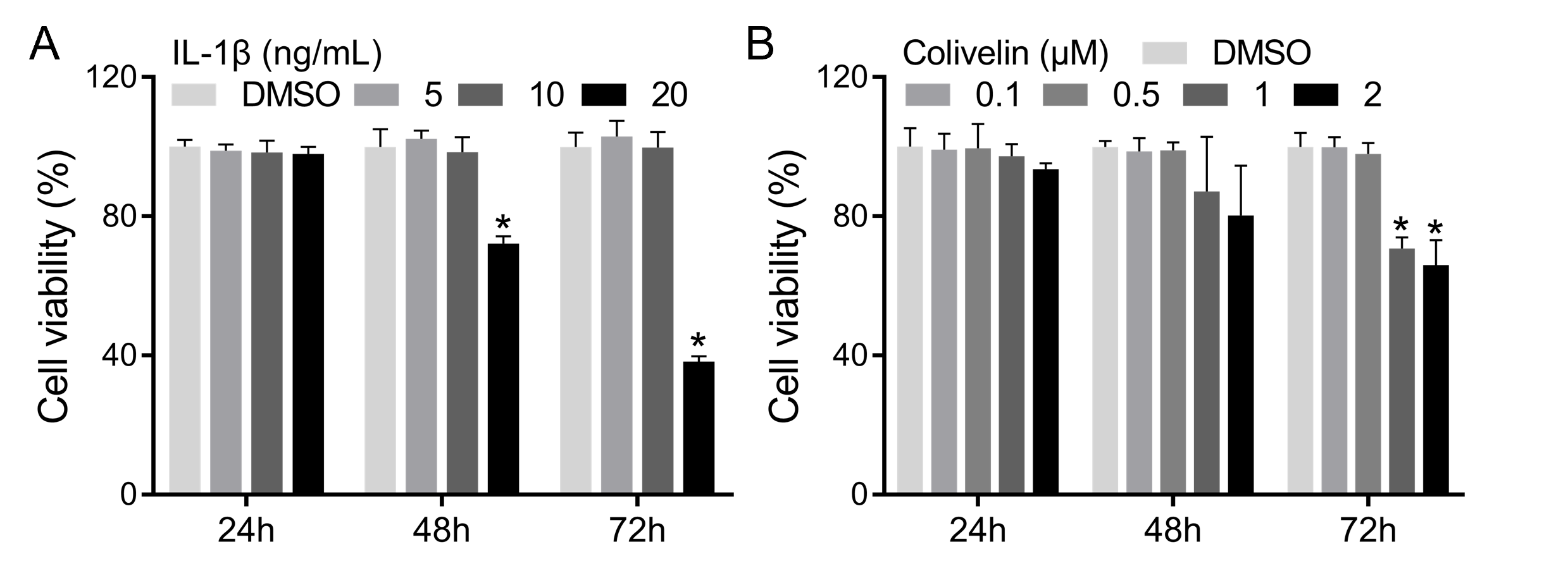


**Fig. S4. Effects of IL-1β and Colivelin on the viability of primary mouse articular chondrocytes.**

(A) Cell viability of primary chondrocytes treated with vehicle (DMSO) or different concentrations of IL-1β (5, 10, 20 ng/mL) for 24, 48 and 72 h, measured by CCK-8 assay. (B) Cell viability of primary chondrocytes treated with vehicle (DMSO) or different concentrations of Colivelin (0.1, 0.5, 1, 2 μM) for 24, 48 and 72 h, measured by CCK-8 assay. Quantitative analyses were based on four biological replicates. ^*^ indicate significant differences between the treatment group and its corresponding vehicle control.


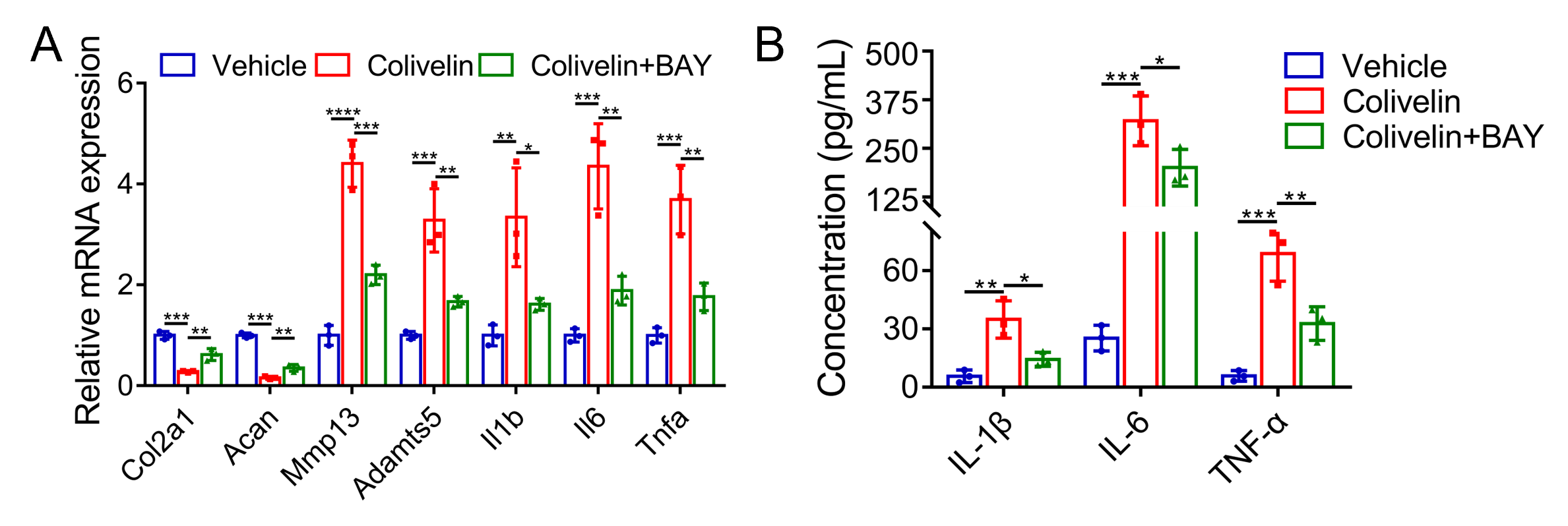


**Fig. S5. Phosphorylated STAT3 regulates ECM metabolism and inflammatory cytokine expression in chondrocytes via the NF-κB pathway.**

(A) RT-qPCR analysis of the mRNA expression of NF-**κ**B target genes in chondrocytes treated with Colivelin and/or BAY (1 μM). (B) ELISA detection of the concentrations of pro-inflammatory cytokines in the culture supernatants of chondrocytes with the same treatments. Quantitative analyses were based on three biological replicates. ^*^*P* < 0.05. ^**^*P* < 0.01. ^***^*P* < 0.001. ^****^*P* < 0.0001.


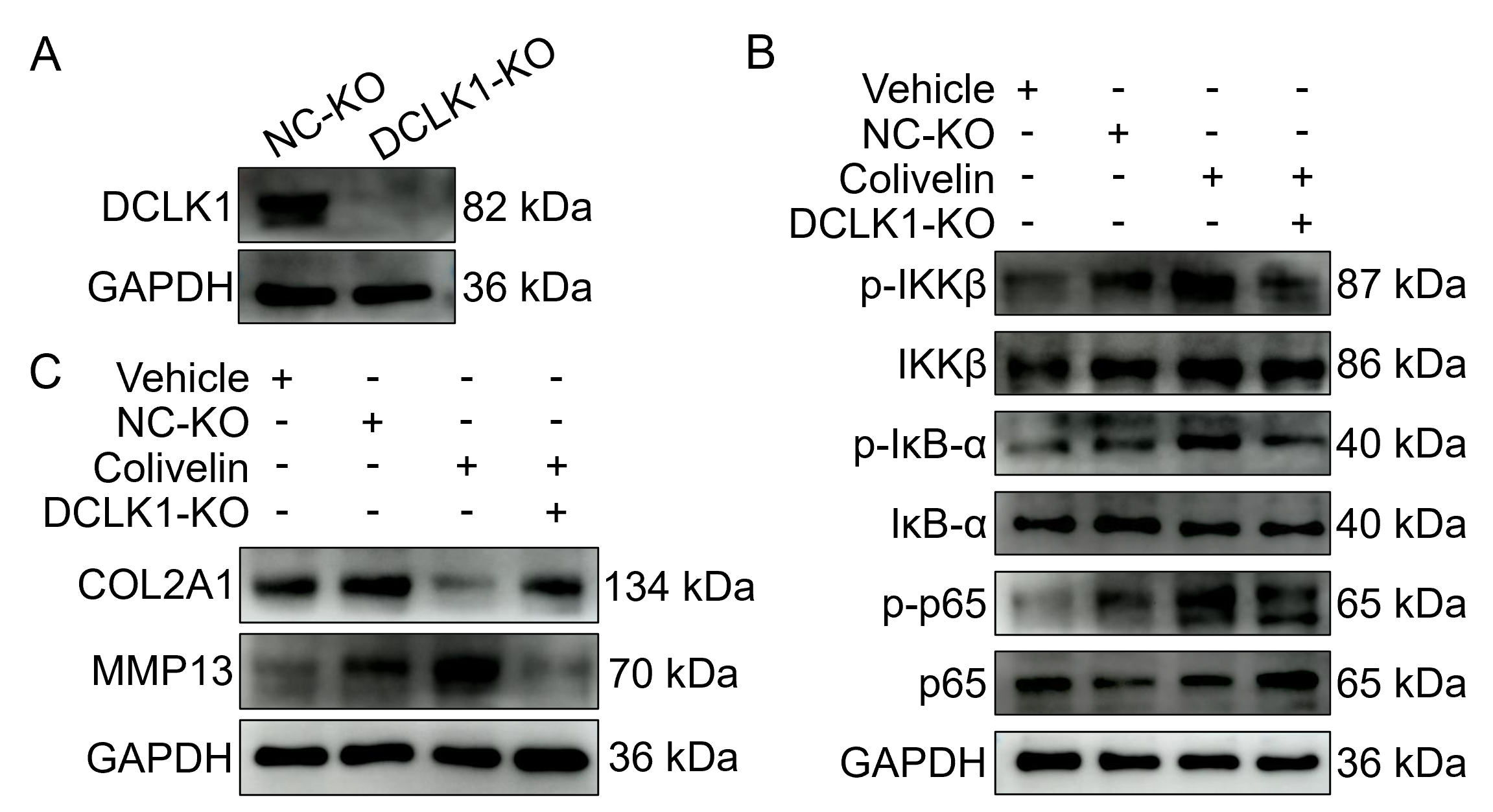


**Fig. S6. CRISPR/Cas9-mediated DCLK1 knockout confirms its specific role in STAT3-induced NF-κB activation and ECM dysregulation.**

(A) Immunoblotting to validate DCLK1 knockout efficiency in chondrocytes transduced with CRISPR/Cas9 lentivirus targeting *Dclk1* (DCLK1-KO) or non-targeting control lentivirus (NC-KO). (B) Immunoblotting analysis of NF-κB pathway activation markers in NC-KO and DCLK1-KO chondrocytes treated with vehicle or Colivelin for 72 h. (C) Immunoblotting analysis of ECM markers in chondrocytes treated as indicated.


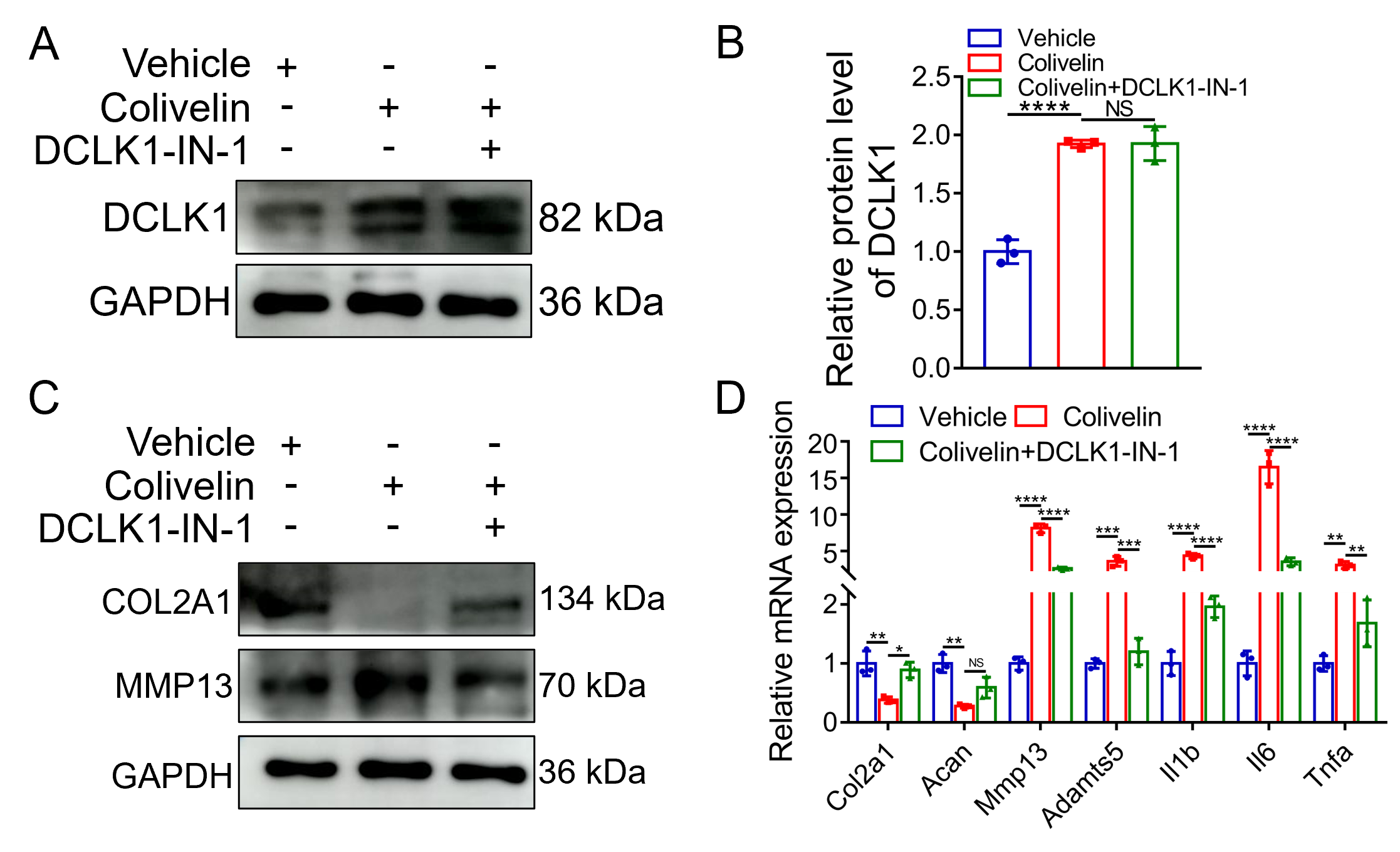


**Fig. S7. DCLK1‑IN‑1 does not alter DCLK1 protein expression but reverses Colivelin‑induced ECM dysregulation in chondrocytes.**

(A, B) Immunoblotting analysis of DCLK1 in chondrocytes treated with Colivelin (0.5 μM) and/or DCLK1-IN-1 (1 μM). The band intensities were quantified and normalized against GAPDH. (C) Representative immunoblots of ECM markers in chondrocytes treated as indicated. (D) RT-qPCR analysis of anabolic (*Col2a1*, *Acan*), catabolic (*Mmp13*, *Adamts5*), and inflammatory (*Il1b*, *Il6*, *Tnfa*) genes. Quantitative analyses were based on three biological replicates. NS, not significant. ^*^*P* < 0.05. ^**^*P* < 0.01. ^***^*P* < 0.001. ^****^*P* < 0.0001.


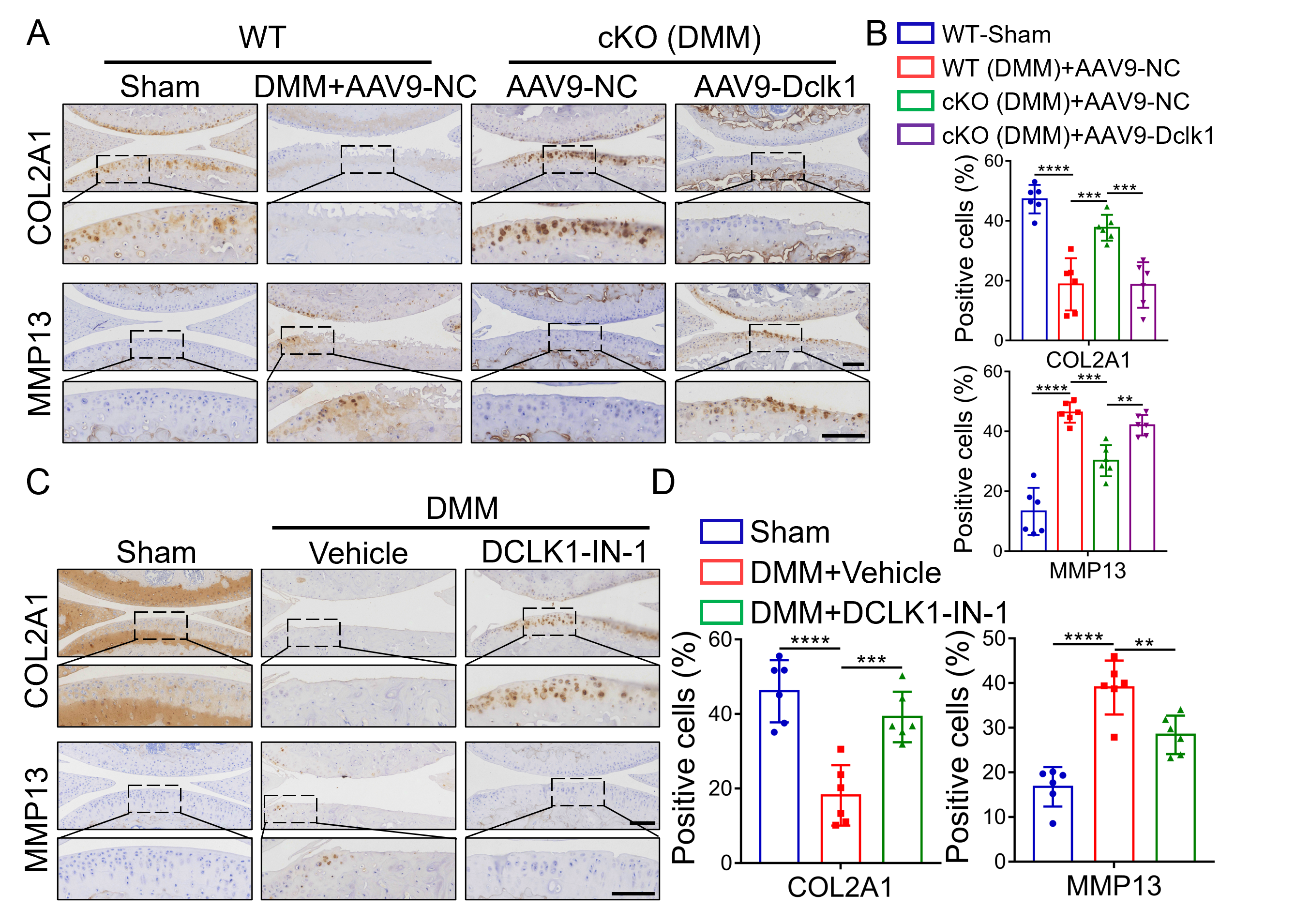


**Fig. S8. Pharmacological inhibition of DCLK1 restores ECM homeostasis in DMM-induced OA mice.**

(A, C) Representative IHC images of COL2A1 and MMP13 in chondrocytes from each group. Scale bar: 100 μm. (B, D) Quantification of COL2A1 and MMP13 expression. Quantitative analyses were based on six biological replicates. ^**^*P* < 0.01. ^***^*P* < 0.0001. ^****^*P* < 0.0001.

**
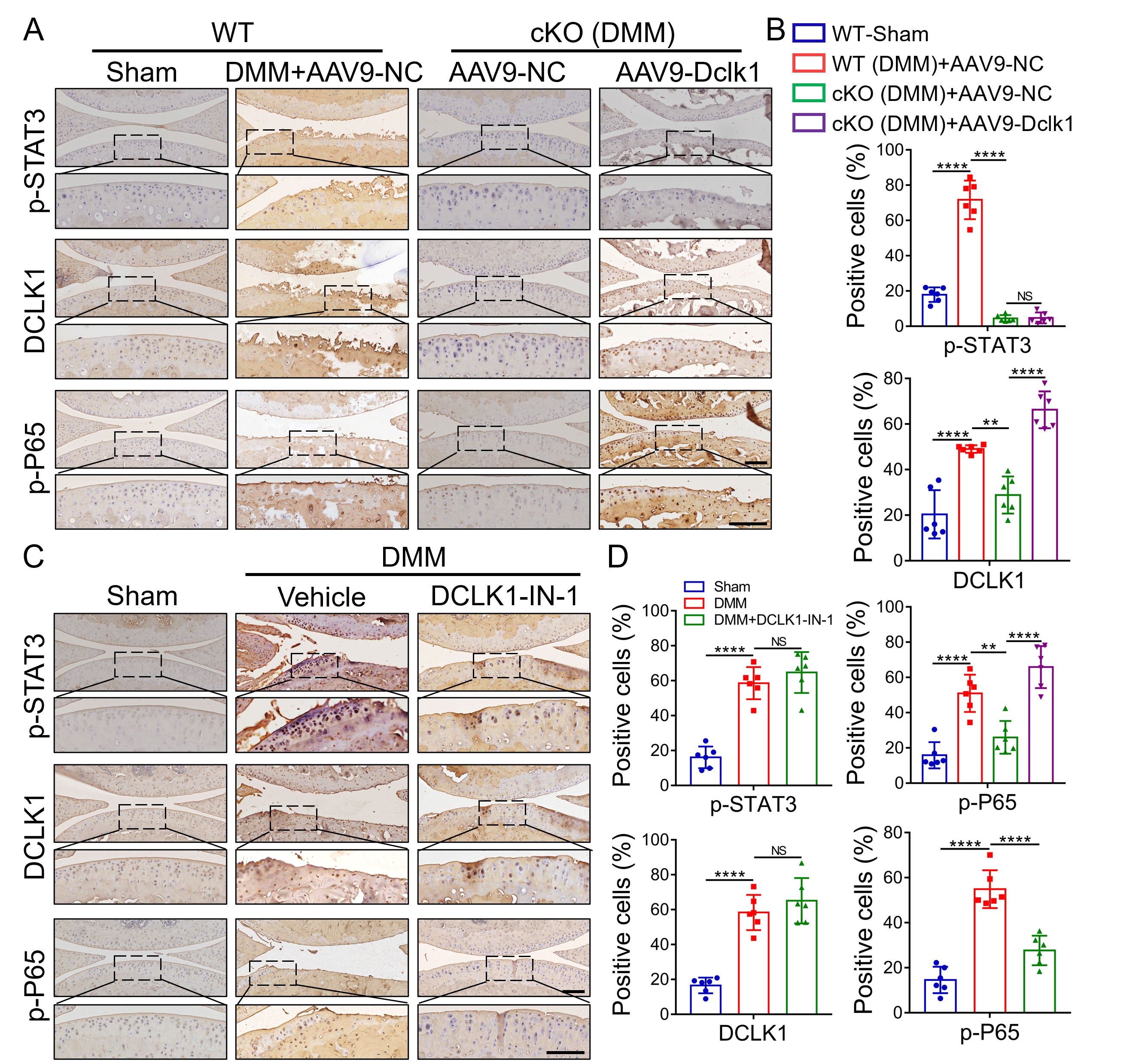
**

**Fig. S9. In vivo validation of the p-STAT3/DCLK1/NF-κB axis by IHC staining.**

(A, C) Representative IHC images of p-STAT3, DCLK1 and p-p65 in chondrocytes from each group. Scale bar: 100 μm. (B, D) Quantification of p-STAT3, DCLK1 and p-p65 expression. Quantitative analyses were based on six biological replicates. NS, not significant. ^**^*P* < 0.01. ^****^*P* < 0.0001.

**
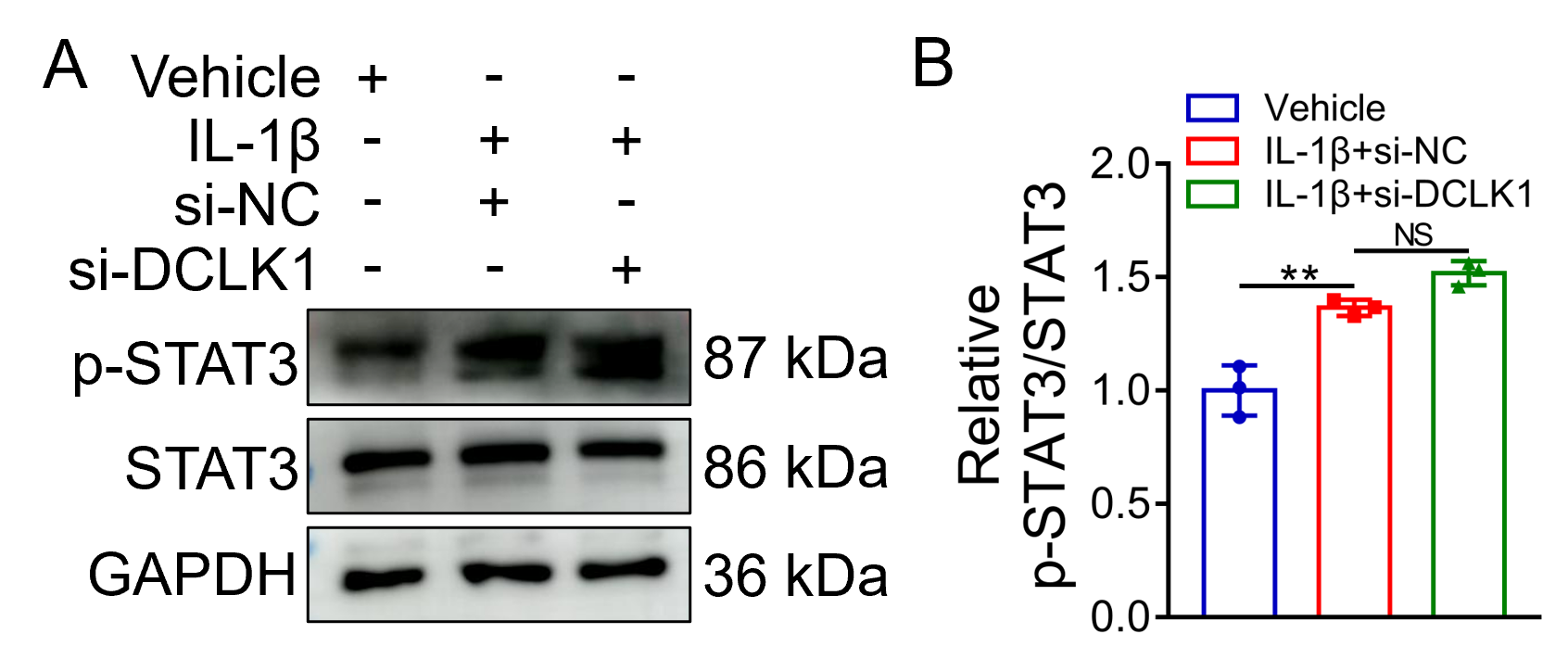
**

**Fig. S10. Effect of DCLK1 knockdown on IL-1β-induced STAT3 phosphorylation in primary mouse chondrocytes.**

(A, B) Representative immunoblotting analysis of p-STAT3 and STAT3 in chondrocytes transfected with si-NC or si-DCLK1, followed by treatment with IL-1β (10 ng/mL). The band intensities were quantified and normalized against GAPDH. Quantitative analyses were based on three biological replicates. NS, not significant. ^**^*P* < 0.01.

**Supplementary Tables**

**Table S1. Detailed information of patients.**

| Case | Gender | Age | Diagnosis |
| --- | --- | --- | --- |
| 1 | Female | 63 | Osteoarthritis |
| 2 | Female | 71 | Osteoarthritis |
| 3 | Male | 72 | Osteoarthritis |
| 4 | Female | 59 | Osteoarthritis |
| 5 | Female | 66 | Osteoarthritis |
| 6 | Female | 60 | Osteoarthritis |
| 7 | Female | 46 | Tibial plateau fracture |
| 8 | Male | 39 | Traumatic cartilage injury |
| 9 | Male | 44 | Tibial plateau fracture |
| 10 | Female | 52 | Comminuted fracture |

**Table S2. Primers for genotyping.**

| Gene | Direction | Primer sequence (5’-3’) |
| --- | --- | --- |
| *Stat3*^fl/fl^ | forward | TTGACCTGTGCTCCTACAAAAA |
|  | reversed | CCCTAGATTAGGCCAGCACA |
| *Col2a1*^CreERT2^ | forward | CACTGCGGGCTCTACTTCAT |
|  | reversed | ACCAGCAGCACTTTTGGAAG |

**Table S3. Primers’ sequences for the quantitative real-time PCR.**

| Gene | Direction | Primer sequence (5’-3’) |
| --- | --- | --- |
| *Dclk1* | forward | TCCACCGGAATTGAACTCGG |
|  | reversed | GGGAGCGAACAGTCTCAGA |
| *Col2a1* | forward | GGGTCACAGAGGTTACCCAG |
|  | reversed | ACCAGGGGAACCACTCTCAC |
| *Acan* | forward | GTGGAGCCGTGTTTCCAAG |
|  | reversed | AGATGCTGTTGACTCGAACCT |
| *Mmp13* | forward | TGTTTGCAGAGCACTACTTGAA |
|  | reversed | CAGTCACCTCTAAGCCAAAGAAA |
| *Adamts5* | forward | CCCAGGATAAAACCAGGCAG |
|  | reversed | CGGCCAAGGGTTGTAAATGG |
| *Il1b* | forward | GAAATGCCACCTTTTGACAGTG |
|  | reversed | TGGATGCTCTCATCAGGACAG |
| *Il6* | forward | CTGCAAGAGACTTCCATCCAG |
|  | reversed | AGTGGTATAGACAGGTCTGTTGG |
| *Tnfa* | forward | CAGGCGGTGCCTATGTCTC |
|  | reversed | CGATCACCCCGAAGTTCAGTAG |
| *Gapdh* | forward | AGGTCGGTGTGAACGGATTTG |
|  | reversed | GGGGTCGTTGATGGCAACA |

**Table S4. The siRNA sequences.**

| siRNA | Discription | Sequence (5’-3’) |
| --- | --- | --- |
| *Stat3*-1 | sense | CUGGAUAACUUCAUUAGCA (dT)(dT) |
|  | antisense | UGCUAAUGAAGUUAUCCAG (dT)(dT) |
| *Stat3*-2 | sense | CAUCAAUCCUGUGGUAUAA (dT)(dT) |
|  | antisense | UUAUACCACAGGAUUGAUG (dT)(dT) |
| *Stat3*-3 | sense | GAAAUUGACCAGCAAUAUA (dT)(dT) |
|  | antisense | UAUAUUGCUGGUCAAUUUC (dT)(dT) |
| *Dclk1*-1 | sense | GCAGCUCAAUGGAUGAGAA (dT)(dT) |
|  | antisense | UUCUCAUCCAUUGAGCUGC (dT)(dT) |
| *Dclk1*-2 | sense | GCAGCAGGAGUUUCUGUAA (dT)(dT) |
|  | antisense | UUACAGAAACUCCUGCUGC (dT)(dT) |
| *Dclk1*-3 | sense | CAGAGAAUCUGCUGGUGUA (dT)(dT) |
|  | antisense | UACACCAGCAGAUUCUCUG (dT)(dT) |

**Table S5. Prioritized candidate p-STAT3 target genes identified by integrated RNA-seq and CUT&Tag-seq.**

| Gene | logFC (KO vs Control) | FDR | Peak Fold Enrichment | Relevance to OA | | Selection Decision |
| --- | --- | --- | --- | --- | --- | --- |
| ***Dclk1*** | -1.40 | 1.18×10⁻¹⁷ | 4.23 | | activate NF-κB; role in OA unknown | **functional validation** |
| *Socs3* | -2.48 | 2.16×10⁻²³ | 5.14 | | Feedback inhibitor of STAT3 | Not; negative feedback regulator |
| *Junb* | -1.65 | 7.91×10⁻¹⁹ | 4.63 | | AP-1 component | Not; downstream of MAPK/JNK |
| *Nfkbia* | -2.71 | 1.19×10⁻²¹ | 4.04 | | NF-κB inhibitor | Not; not a pathway activator |
| *Adamts1* | -1.61 | 1.46×10⁻²¹ | 6.59 | | known NF-κB target | Not; not an upstream NF-κB activator |
| *Adamts5* | -2.42 | 9.42×10⁻²⁵ | 2.92 | | ECM degradation enzyme | Not; distal regulation unclear |
| *Mmp3* | -9.68 | 2.09×10⁻²⁰ | 4.44 | | ECM degradation enzyme | Not; very low basal expression |
| *Ptgs2* | -4.19 | 8.18×10⁻²⁵ | 3.26 | | inflammation | Not; peak not at promoter |
| *Il6* | -9.28 | 7.36×10⁻¹⁵ | 6.24 | | Proinflammatory cytokine | Not; downstream of NF-κB |
| *Ccl2* | -7.16 | 4.95×10⁻²⁹ | 3.51 | | monocyte recruitment | Not; peak not at promoter |

The selection followed three criteria: (1) significant differential expression upon STAT3 knockout; (2) presence of a strong p-STAT3 binding peak in the promoter region (Fold Enrichment > 4); and (3) established or plausible relevance to OA pathology, particularly in inflammation or ECM metabolism.
